# Specifically Increased Rate of Infections in Children Post Measles in a High Resource Setting

**DOI:** 10.3389/fped.2022.896086

**Published:** 2022-06-09

**Authors:** Daniel Bühl, Olga Staudacher, Sabine Santibanez, Rainer Rossi, Hermann Girschick, Volker Stephan, Beatrix Schmidt, Patrick Hundsdoerfer, Arpad von Moers, Michael Lange, Michael Barker, Marcus A. Mall, Ulrich Heininger, Dorothea Matysiak-Klose, Annette Mankertz, Horst von Bernuth

**Affiliations:** ^1^Department of Pediatric Respiratory Medicine, Immunology and Critical Care Medicine, Charité—Universitätsmedizin Berlin, Corporate Member of Freie Universität Berlin, Humboldt-Universität zu Berlin, Berlin, Germany; ^2^Department of Immunology, Labor Berlin GmbH, Berlin, Germany; ^3^National Reference Center for Measles, Mumps, Rubella, Robert Koch Institute, Berlin, Germany; ^4^Department of Pediatrics, Vivantes Klinikum Neukölln, Berlin, Germany; ^5^Children’s Hospital, Vivantes Klinikum im Friedrichshain, Berlin, Germany; ^6^Department of Pediatrics, Sana Klinikum Lichtenberg, Berlin, Germany; ^7^St. Joseph’s Center for Pediatric and Adolescent Medicine, St. Joseph Krankenhaus, Berlin, Germany; ^8^Department of Pediatric and Adolescent Medicine, Helios-Klinikum Berlin-Buch, Berlin, Germany; ^9^Department of Pediatrics and Neuropediatrics, DRK Kliniken Berlin Westend, Berlin, Germany; ^10^Department of Pediatrics, Evangelisches Waldkrankenhaus Spandau, Berlin, Germany; ^11^Department of Pediatrics, Helios Klinikum Emil von Behring, Berlin, Germany; ^12^Berlin Institute of Health at Charité—Universitätsmedizin Berlin, Berlin, Germany; ^13^German Center for Lung Research (DZL), Berlin, Germany; ^14^Infectious Diseases and Vaccinology, University of Basel Children’s Hospital, Basel, Switzerland; ^15^Department for Infectious Disease Epidemiology, Immunization Unit, Robert Koch Institute, Berlin, Germany; ^16^Berlin-Brandenburg Center for Regenerative Therapies (BCRT), Berlin, Germany

**Keywords:** measles, outbreak, immune amnesia, Europe, high resource setting

## Abstract

**Objectives:**

Post-measles increased susceptibility to subsequent infections seems particularly relevant in low-resource settings. We tested the hypothesis that measles causes a specifically increased rate of infections in children, also in a high-resource setting.

**Methods:**

We conducted a retrospective cohort study on a large measles outbreak in Berlin, Germany. All children with measles who presented to hospitals in Berlin were included as cases, children with non-infectious and children with non-measles infectious diseases as controls. Repeat visits within 3 years after the outbreak were recorded.

**Results:**

We included 250 cases, 502 non-infectious, and 498 infectious disease controls. The relative risk for cases for the diagnosis of an infectious disease upon a repeat visit was 1.6 (95% CI 1.4–2.0, *p* < 0.001) vs. non-infectious and 1.3 (95% CI 1.1–1.6, *p* = 0.002) vs. infectious disease controls. 33 cases (27%), 35 non-infectious (12%) and 57 (18%) infectious disease controls presented more than three times due to an infectious disease (*p* = 0.01, and *p* = 0.02, respectively). This results in a relative risk of more than three repeat visits due to an infection for measles cases of 1.8 (95% CI 1.3–2.4, *p* = 0.01), and 1.4 (95% CI 1.0–1.9, *p* = 0.04), respectively.

**Conclusion:**

Our study demonstrates for the first time in a high-resource setting, that increased post-measles susceptibility to subsequent infections in children is measles-specific—even compared to controls with previous non-measles infections.

## Introduction

Measles causes significant mortality and morbidity in children (1, 2). In 2019 the WHO estimated 9,828,400 measles cases with 207,500 associated deaths worldwide, mostly affecting children under the age of 5 years (3). In high-resource regions, vaccine hesitancy remains the major risk for outbreaks and thus is a declared threat to global health (4–8). Neither Europe, nor the United States have met the WHO goal of more than 95% of the population being vaccinated with two doses against measles according to the nationally recommended schedule (3, 9, 10). Measles was assumed to cause short-term immunosuppression as early as 1765 by Francis Homes and 1908 by Clemens von Pirquet (11). The first observed a higher susceptibility to diphtheria, the latter the loss of cutaneous reactivity to tuberculin post measles (11, 12). However, this loss of delayed hypersensitivity as measured by skin reaction to tuberculin has also been reported in humans infected with influenza, varicella and polio viruses (13, 14). In 2012 immunosuppression post measles was coined “measles-induced immune amnesia” (15).

There are three major lines of evidence for measles-induced immune amnesia: First, studies at population level demonstrated an unexpectedly large reduction of all-cause mortality and morbidity in children after introduction of measles vaccination (16–18). This reduction exceeded the expected avoidance of directly measles-related sequelae, and is attributed to a general reduction of infections due to assumed unspecific effects of the vaccination (16–18). Second, 7 weeks post measles, significant skewing of the preexisting diversity of antibody repertoires and a shift to immature B cell-receptor repertoires was observed in a cohort of Dutch children with low vaccination coverage (19–21). A similar skewing of the B cell-receptor repertoire along with a strong skewing of the memory T cell pool were observed in macaques upon experimental infections with measles, attributed to the infection and depletion of CD150-positive immune cells (15, 19, 20, 22, 23). These studies identified two phases of immunologic effects: A short-term immunosuppression in the first weeks after measles-infection and a longer lasting susceptibility to infections of up to 5 years (18, 24–27). Third, one ecological and three epidemiological studies observed an increased susceptibility to infections after measles (24–27).

Yet, studies that compare clinical effects post measles on the population level with the one after other infections remain scarce. Taking advantage of a cohort of children in the largest geographically and temporally confined measles outbreak in Germany in the past 15 years, we tested for the first time the hypothesis that measles is associated with a specifically increased rate of infections also in a high-resource setting (28–33). To test for a specific effect of measles we compared the rate of infections in children post measles not only with the one in children without measles but also with the rate of infections in children post non-measles infections. Since sequela post measles are mostly observed in children, we conducted a retrospective cohort study to estimate the relative risk for subsequent infectious diseases in children. The observational period was set to 3 years as measles-induced immune amnesia waned after 26 months, 30 months and 1 year, respectively, in previous studies in high resource settings (24, 26, 27).

To our knowledge, this is the first investigation that compares the number of infections post measles not only with the number of infections in children who had not contracted measles, but also in children who had contracted other, non-measles infections. Taking advantage of a single geographically and temporally confined measles outbreak allowed us to follow on the specific impact of measles on morbidity among children in a genetically and socially highly diverse population in a high resource setting.

## Materials and Methods

### Study Design

The study was approved by the ethics committee of the Charité—Universitätsmedizin Berlin (EA2_028_20). The retrospective cohort study was conducted in all nine children’s hospitals in Berlin. Children (age 18 years) who fulfilled the WHO case definition for measles and presented to any emergency room during the measles epidemic between 20th October 2014 and 30th August 2015 were included as cases (28, 29, 31). “Laboratory confirmed” measles was defined as a laboratory confirmed infection. “Clinically compatible” measles was defined by the presence of the following measles-defining symptoms: fever, rash and cough, coryza or conjunctivitis. “Suspected” measles was defined by the presence of two out of three measles-defining symptoms. Patients with chronic diseases (e.g., asthma, diabetes, cancer, etc.) were excluded, as well as children with permanent residency outside Berlin.

### Study Population

The outbreak in the Berlin area between the 20th October 2014 and the 30th August 2015 comprised 1,344 patients with measles, of whom 720 were children (29, 31). Measles strain D8-Rostov on Don-2987 was imported from Serbia to Germany, and spread among the Berlin population (29, 31). In 2014/2015, the population of Berlin comprised 3,562,166 inhabitants with diverse and heterogeneous ancestry [e.g., 573,342 (16%) non-German citizens and 444,257 (12.5%) German citizens with a migrant background from over 190 countries], 538,326 (15%) of whom where under the age of 18 (28, 34).

### Controls Comprise Children Who Presented for Non-infectious Reasons, and for Non-measles Infections

**FIGURE 1:**
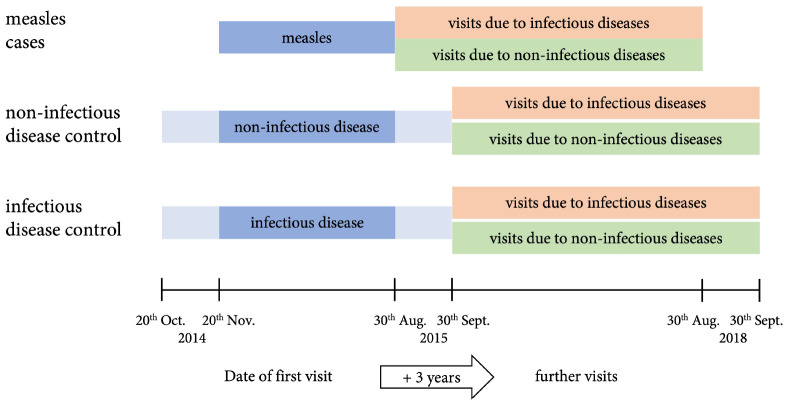
Study design. Children and adolescents (age ≤ 18 years) who fulfilled the WHO case definition for measles and presented to the emergency room during the measles epidemic between 20th October 2014 and 30th August 2015 were included as cases. Controls were matched for sex, age (± 10), date of first visit to the hospital (±1 month, illustrated), and duration of in-patient care if applicable (± 20% in full days). All patients were followed up for 3 years.

For each patient with measles four controls were identified: two who presented with an acute non-infectious disease and two further controls with an acute infectious, non-measles disease (Supplementary Table 1). Controls were matched for sex, age (± 10%), date of first visit to the hospital (±1 month), and duration of in-patient care if applicable (± 20% in full days). The age of controls was chosen in a relative interval of ± 10% of the age of cases, because a relative interval reflects differences in growth and development among different age groups more realistically than a fixed time span. Study design is illustrated in Figure 1. Children with chronic diseases or measles before or during the follow-up period were excluded. If less than four controls could be identified by these criteria, the search was first broadened to any sex (in 114 controls matching 64 cases), second to a broader range of the first visit ± 2 months (in 113 controls matching 70 cases) and last to any length of stay in the hospital (in 25 controls matching 15 cases) (details are shown in Supplementary Table 2).

### Data Source

The following data were retrieved from the hospitals’ patient management systems: Age (years and months) at first visit, sex, postal code of residence, asylum status (if documented), hospital, date of admission, date of discharge, primary ICD-10-diagnosis, stay on intensive care unit (ICU) (yes/no), measles immunization status, WHO case definition for measles and all repeat visits to the emergency room during the following 3 years (regardless whether patients remained outpatients or were admitted). The information outlined above was equally recorded for repeat visits. The ICD-10-diagnoses of repeat visits were grouped as infectious or non-infectious (Supplementary Table 1). Repeat visits directly related to the previous one were disregarded: Related visits due to non-infectious diagnoses were identified by the same ICD-10-code. Related visits due to infectious diagnoses were clustered into body regions and assigned an approximate duration of infection in which a visit with an ICD-10-code from the same cluster was most likely related (Supplementary Table 1). Calculations on repeat visits due to infectious and non-infectious diseases were calculated among patients and controls with repeat visits.

### Statistical Analysis

IBM SPSS Statistics 27 and R (Version 4.0.4.) were used to perform statistical tests. Proportions were compared by (two sided) two proportion z-tests. One-sided *p* values were calculated using Fishers Exact or the Mann–Whitney *U* test. Risk Ratios (RRs) are reported with a confidence interval (CI) of 95%. The comparability of study and outbreak populations was calculated by Kolmogorov-Smirnov test for age distribution and Chi-Square test for residential district.

## Results

### Composition of Study Population

We identified 262 children with measles who presented to an emergency room of the nine children hospitals in Berlin during the 2014/2015 outbreak. This comprises 36.4% of the total of 720 children reported with measles during this outbreak (31). 12 had to be excluded because of a chronic disease (*n* = 9), permanent residence outside of Berlin (*n* = 2), or death related to measles (*n* = 1). Baseline characteristics of cases are shown in Table 1. 182 cases (73%) were categorized as “laboratory confirmed,” 65 (26%) as “clinically compatible” and 3 (1%) as “suspected” according to the WHO classification. The distribution of cases per district of Berlin is shown in Supplementary Figure 1. Comparison of age distribution, proportion of asylum seekers and residential districts yielded no significant differences between study cases and the group of all children affected by measles in Berlin (*p* = 0.05, *p* = 0.24, *p* = 0.92, respectively—Supplementary Figures 2, 3). For each case four controls were identified, resulting in a total of 502 children with a non-infectious diagnosis and 498 with an infectious diagnosis at the time of inclusion, as two patients had to be reassigned from the infectious to the non-infectious disease control group later in the process. The most frequent diagnoses among non-infectious disease controls on first visit were concussions and fractures, while infectious disease controls mainly presented with acute upper respiratory infections, gastroenteritis, bronchitis, and otitis media caused by unspecified pathogens. Detailed characteristics of controls are summarized in Table 1.

**TABLE 1:** Baseline characteristics.

		Measles cases (*n* = 250)	Non-infectious disease controls (*n* = 502)	Infectious disease controls (*n* = 498)
**Variable**	**Category**			
Sex, n (%)	Male	136 (54)	291 (58)	280 (56)
	Female	114 (46)	211 (42)	218 (44)
Age, median (Quartile 1, Quartile 3)		2.8 (0.9, 10.7)	2.7 (0.8, 10.8)	2.5 (0.9, 10.7)
Documented asylum status n (%)		44 (18)	6 (1)	7 (1)
Type of admission, n (%)	Outpatient	154 (62)	310 (62)	307 (62)
	Inpatient	96 (38)	192 (38)	191 (38)
	Duration of stay, mean (SD)	3.9 (2.3)	3.0 (1.9)	3.6 (1.9)
WHO measles case classification, n (%)	Laboratory confirmed	182 (73)		
	Clinically compatible	65 (26)		
	Suspected	3 (1)		
MMR-vaccination status, n (%)	Not vaccinated	153 (61)	95 (19)	108 (22)
	1x	18 (7)	9 (2)	26 (5)
	2x	8 (3)	42 (8)	80 (16)
	Unknown	71 (28)	356 (71)	284 (57)
Site of first visit, n (%)	Hospital A	11 (4)	22 (4)	22 (4)
	Hospital B	32 (13)	52 (10)	65 (13)
	Hospital C	72 (29)	248 (49)	217 (44)
	Hospital D	19 (8)	0 (0)	0 (0)
	Hospital E	26 (10)	41 (8)	49 (10)
	Hospital F	58 (23)	112 (22)	111 (22)
	Hospital G	3 (1)	3 (1)	4 (1)
	Hospital H	14 (6)	0 (0)	0 (0)
	Hospital I	12 (5)	18 (4)	24 (5)

### Cases and Controls With Repeat Visits

Totally 124 (50%) cases, 285 (57%) non-infectious disease controls and 311 (62%) infectious disease controls presented to an emergency room at least once again during the observation period of 3 years and accounted for 311, 680 and 842 repeat visits, respectively. The number of repeat visits due to infections per patient is shown in Supplementary Figure 4. A detailed description of patients with and the respective number of repeat visits is depicted in Tables 2, 3.

**TABLE 2:** Patients with repeat visits to the emergency room.

	Measles cases (*n* = 250)	Non-infectious disease controls (*n* = 502)	Infectious disease controls (*n* = 498)
Patients, n (%)	124 (50)	285 (57)	**311 (62)^a^**
Infectious diagnosis, n (%)	90 (73)	**166 (58)^a^**	221 (71)
Outpatient, n (%)	79 (64)	**152 (53)^a^**	192 (62)
Inpatient, n (%)	19 (15)	**24 (8)^a^**	56 (20)
With ≥ 3 visits	33 (27)	**35 (12)^a^**	**57 (18)^a^**
Non-infectious diagnosis, n (%)	76 (61)	**220 (77)^b^**	220 (71)
Outpatient, n (%)	69 (56)	**199 (70)^a^**	196 (63)
Inpatient, n (%)	13 (11)	43 (15)	42 (14)

*Number of patients with repeat visits in the 3 years follow-up period, divided into groups by type of diagnosis (infectious, non-infectious), and type of admission (outpatient, inpatient).*

*Percentages add up to > 100% because some patients had multiple visits.*

*^a^p < 0.05, ^b^p < 0.001, comparing the proportion of patients to cases. Bold values show significant differences.*

**TABLE 3:** Number of repeat visits to the emergency room.

	Measles cases (*n* = 250)	Non-infectious disease controls (*n* = 502)	Infectious disease controls (*n* = 498)
Repeat visits, n	334	680	843
Infectious diagnosis, n (%)	212 (64)	**311 (46)^b^**	**464 (55)^a^**
Outpatient, n (%)	190 (57)	**286 (42)^b^**	**398 (47)^a^**
Inpatient, n (%)	22 (7)	**25 (4)^a^**	66 (8)
Non-infectious diagnosis, n (%)	122 (37)	**369 (54)^b^**	**379 (45.)^a^**
Outpatient, n (%)	106 (32)	**320 (47)^b^**	**332 (39)^a^**
Inpatient, n (%)	16 (5)	49 (7)	47 (5)

*Number of repeat visits in the 3 years following inclusion, divided into groups by type of diagnosis (infectious, non-infectious) and type of admission (outpatient, inpatient).*

*^a^p < 0.05, ^b^p < 0.001, comparing the proportion of repeat visits to cases. Bold values show significant differences.*

Most common infectious diagnoses throughout all groups were acute upper respiratory infections, gastroenteritis, unspecified viral infection, bronchitis, otitis media, pharyngitis and laryngitis, while most common non-infectious diagnoses were concussion, superficial head injury, subluxation of radial head, nausea and vomiting. While diagnoses were broadly distributed, not showing an increase on infections in specific regions, the overall quantity and proportions showed significant differences.

### Reasons for Repeat Visits: More Repeated Visits Due to Infections Among Cases Than Among Non-infectious Disease Controls

The proportion of patients who presented to an emergency room during the follow up interval was comparable in cases and non-infectious disease controls (124 (50%) cases vs. 285 (57%) controls, *p* = 0.08). When stratified by diagnosis given upon a repeat visit, significantly more cases with repeat visits returned because of an infectious disease [90 (73%) cases vs. 166 (58%) controls, *p* = 0.006] and were more frequently admitted (19 (15%) cases vs. 24 (8%) controls, *p* = 0.03). We observed the same distribution of diagnoses at repeat visits when analyzing the total number of visits instead of patients (see Table 3).

### Reasons for Repeat Visits: More Repeated Visits Due to Infections Among Cases Than Among Infectious Disease Controls

Also, when comparing cases to infectious disease controls, differences were present. The proportion of patients with any repeat visit to the hospital was significantly higher in controls [124 (50%) cases vs. 311 (62%) controls, *p* = 0.001]. However, there was no significant difference when these patients were stratified by diagnoses at repeat visits. Though, when analyzing the number of visits per diagnosis category, the proportion of infectious diagnoses was significantly higher in cases [212 (64%) visits in cases vs. 464 (56%) in controls, *p* = 0.01] (see Tables 2, 3).

### Relative Risks for Subsequent Infections Are Increased After Measles

**FIGURE 2:**
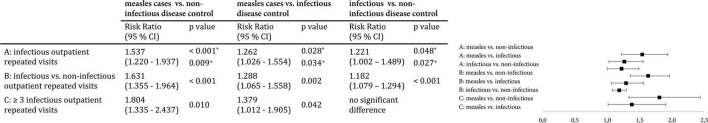
Risk ratio for repeat visits to the emergency room due to infections. (A) Relative risk of cases for an outpatient repeat visit with an infectious diagnosis, calculated using the Man-Whitney-U test (regarding only patients with repeat visits). (B) Relative risk of cases for an outpatient repeat visit with an infectious diagnosis compared to a non-infectious diagnosis, calculated using the Chi-Square Test. (C) Relative risk of cases for three or more infectious outpatient repeat visits, calculated using the Chi-Square Test.

Children post measles who were seen in the emergency room had a risk ratio of 1.6 (95% CI 1.4–2.0, *p* < 0.001) for being diagnosed with an infection compared to non-infectious disease controls and a risk ratio of 1.3 (95% CI 1.1–1.6, *p* = 0.002) compared to infectious disease controls. When comparing the two control groups, infectious disease controls had an increased risk of 1.2 (95% CI 1.1–1.3, *p* < 0.001) for being diagnosed with an infection compared to non-infectious disease controls. Additionally, patients who presented more than three times due to an infectious disease during follow-up accounted for a significantly higher proportion in cases [33 (27%) cases, 35 (12%) non-infectious, *p* < 0.001, and 57 (18%) infectious disease controls, *p* = 0.04], with an elevated risk ratio for cases of 1.8 (95% CI 1.3–2.4, *p* = 0.01) compared to non-infectious controls and 1.4 (95% CI 1.0–1.9, *p* = 0.04) compared to infectious controls (see Figure 2).

## Discussion

The Berlin measles outbreak in 2014/2015 constitutes the largest outbreak in Germany during the past 15 years. It offered the unique opportunity to test the hypothesis that there is a specifically increased rate of infections post measles, compared to effects post other infections, also in high-resource settings. To our knowledge, our results indicate for the first time an elevated relative risk of repeat visits to the emergency room due to infections for children who contracted measles (cases) compared to not only non-infectious (relative risk of 1.6) but also infectious disease controls (relative risk of 1.3). To our knowledge, these data are also the first to demonstrate an increased risk for infections after a geographically as well as temporally confined measles outbreak in a high-resource setting among a genetically and socially highly diverse population.

In the United Kingdom 2,228 children post measles had been investigated through population based data between 1990 and 2014 (26). During a maximum of 5 years follow-up there was an increased relative risk for infections in children after measles compared to children free of measles (26). This relative risk decreased but remained significant, from 1.44 in the first month to 1.15, between 2.5 and 5 years of follow-up (26). Even within our by far smaller sample size of 250 cases we found a comparably increased relative risk for infectious diseases after measles. In 2020, a Swiss study reported on an increased rate of rehospitalization due to infectious diseases for children in the first year who had been hospitalized for measles between 2000 and 2015 (27). The authors found a higher relative risk of 5.2 for rehospitalization due to infections in the first year post measles compared to non-infectious controls (27). However, these results were based on 113 cases only and two non-infectious disease controls per case, with a total of twelve and six children who were readmitted to the hospital, respectively (27). In contrast, within our sample of 250 cases, we saw an increased susceptibility to infections post measles over the whole period of 3 years. Also, we included all repeat visits to the emergency room in our analysis, regardless of the patient’s admission to hospital. This allowed a more realistic statistical estimation of the relative risk for further infections. Additionally, we noticed an increased risk ratio for repeat visits due to infections after measles not only compared to non-infectious, but also to infectious disease controls, which was also higher than the risk ratio of infectious compared to non-infectious disease controls, indicating a measles-specific effect on further infections. To our knowledge, no previous study included children with other, non-measles infections as controls. While at least 61% of cases were not vaccinated against measles (documentation in the hospitals’ patient management systems was not complete with respect to the vaccination status), vaccination history was hardly ever recorded in controls. All infectious diseases among cases and controls were not vaccine-preventable, with the exception of one visit in each group. So differences between cases and controls cannot be attributed to general differences in the vaccination history. A further strength of our measles cohort is its heterogeneous composition (28, 31, 34). Compared to the detailed studies on T and B cell immunity post measles, based on an ecological sample of socially and geographically highly clustered Dutch children of a community of orthodox protestants, the diverse composition of our sample makes a shallow gene pool as a possible confounder for an increased susceptibility to infections post measles highly unlikely. By including all patients who presented with measles to one of the nine children’s hospitals of Berlin, we were able to comprise 35% of all known children infected with measles during the outbreak (31). We found no significant differences in age, documented asylum seekers and residence district between our sample and the city’s population that contracted measles at that time.

Germany mainly provides primary pediatric care in private practices and usually only during off hours by emergency rooms in hospitals. Keeping this in mind, our study design has several limitations. First, the sample size was limited to 250 children with measles. Yet, despite this rather small size [at least compared to the United Kingdom-study (26)], differences in repeat visits for infectious diseases and the relative risk for subsequent infections compared to controls were significant. Second, we were not able to collect data on how many repeat visits occurred to pediatricians in private practice. Third, we were not able to identify patients that sought emergency care for any illness following a measles infection diagnosed outside a hospital. Forth, we must acknowledge that more patients among measles cases were asylum seekers than among controls. It is plausible that these children relocated to places outside Berlin during the 3 years of follow-up after the measles outbreak, adding to the percentage of cases lost to follow-up. Additionally, we found a significantly higher proportion of patients with at least one repeat visit due to non-infectious diagnoses in both control groups, compared to cases. Since the incidence of non-infectious symptoms should be the same in cases and controls, it seems at least possible (if not likely) that we lost a bigger proportion of patients to follow-up in the measles group. Last, we were not able to show a shift toward more severe subsequent infections after measles compared to controls. Although few severe, life-threatening and even deadly infections post measles had been observed in Berlin during or shortly after the outbreak on the individual level, there was no significant increase of severe infections post measles on the population level (28). Additionally, although the control groups show similar patient characteristics and no significant difference in visits due to non-infectious diseases, we recorded significantly more visits due to an infection in the infectious disease compared to the non-infectious disease control group. We assume that the included infectious disease controls might display further undocumented risk factors making these patients more susceptible to infections (or at least to repeat visits due to infections). Even so, our study demonstrates for the first time that measles cases are at significantly increased risk for subsequent infections, also if compared to controls post other infectious diseases.

Our findings underline the hypothesis that measles is associated with a specifically increased rate of infections in children, even in a high-resource setting like Germany. While measles-induced immune amnesia is considered to cause measles-related mortality particularly in low-resource settings, our study suggests increased post-measles morbidity in children in any country, regardless of medical resources. Our results reinforce the necessity to overcome vaccine hesitancy. Vaccination of > 95% of the population against measles with two doses will not only expedite the rather abstract WHO long-term and global goal to eliminate measles, but immediately lower local measles related all-cause morbidity.

## Data Availability Statement

The raw data supporting the conclusions of this article will be made available by the authors, without undue reservation.

## Author Contributions

DB and HB designed the study, had full access to all data in the study and take responsibility for the integrity of the data and the accuracy of the data analysis, and wrote the manuscript. DB collected the data. DB, OS, and HB analyzed the data. HG, RR, VS, BS, PH, AMo, ML, and MB provided access to the hospital patient management systems. SS and AMa provided access to laboratory data. UH, DM-K, HG, RR, SS, AMa, MB, and MM critically revised the manuscript. UH initially proposed to investigate the Berlin measles outbreak of 2014/2015. All authors approved the final manuscript as submitted and agreed to be accountable for all aspects of the work.

## Conflict of Interest

HB is an associated member of the standing committee on vaccination against measles at the Robert Koch Institute. DM-K is a member of the standing committee on vaccination against measles at the Robert Koch Institute. AMa and SS are consulting members of the standing committee on vaccination at the Robert Koch Institute. UH is a member of the standing committee on vaccination at the Robert Koch Institute. The remaining authors declare that the research was conducted in the absence of any commercial or financial relationships that could be construed as a potential conflict of interest.

## Publisher’s Note

All claims expressed in this article are solely those of the authors and do not necessarily represent those of their affiliated organizations, or those of the publisher, the editors and the reviewers. Any product that may be evaluated in this article, or claim that may be made by its manufacturer, is not guaranteed or endorsed by the publisher.
