# Corrigendum: Specifically increased rate of infections in children post measles in a high resource setting

**DOI:** 10.3389/fped.2022.1005990

**Published:** 2022-09-08

**Authors:** Daniel Bühl, Olga Staudacher, Sabine Santibanez, Rainer Rossi, Hermann Girschick, Volker Stephan, Beatrix Schmidt, Patrick Hundsdoerfer, Arpad von Moers, Michael Lange, Michael Barker, Marcus A. Mall, Ulrich Heininger, Dorothea Matysiak-Klose, Annette Mankertz, Horst von Bernuth

**Affiliations:** ^1^Department of Pediatric Respiratory Medicine, Immunology and Critical Care Medicine, Charité—Universitätsmedizin Berlin, Corporate Member of Freie Universität Berlin, Humboldt-Universität zu Berlin, Berlin, Germany; ^2^Department of Immunology, Labor Berlin GmbH, Berlin, Germany; ^3^National Reference Center for Measles, Mumps, Rubella, Robert Koch Institute, Berlin, Germany; ^4^Department of Pediatrics, Vivantes Klinikum Neukölln, Berlin, Germany; ^5^Children's Hospital, Vivantes Klinikum im Friedrichshain, Berlin, Germany; ^6^Department of Pediatrics, Sana Klinikum Lichtenberg, Berlin, Germany; ^7^St. Joseph's Center for Pediatric and Adolescent Medicine, St. Joseph Krankenhaus, Berlin, Germany; ^8^Department of Pediatric and Adolescent Medicine, Helios-Klinikum Berlin-Buch, Berlin, Germany; ^9^Department of Pediatrics and Neuropediatrics, DRK Kliniken Berlin Westend, Berlin, Germany; ^10^Department of Pediatrics, Evangelisches Waldkrankenhaus Spandau, Berlin, Germany; ^11^Department of Pediatrics, Helios Klinikum Emil von Behring, Berlin, Germany; ^12^Berlin Institute of Health at Charité—Universitätsmedizin Berlin, Berlin, Germany; ^13^German Center for Lung Research (DZL), Berlin, Germany; ^14^Infectious Diseases and Vaccinology, University of Basel Children's Hospital, Basel, Switzerland; ^15^Department for Infectious Disease Epidemiology, Immunization Unit, Robert Koch Institute, Berlin, Germany; ^16^Berlin-Brandenburg Center for Regenerative Therapies (BCRT), Berlin, Germany

**Keywords:** measles, outbreak, immune amnesia, Europe, high resource setting

In the published article, there was an error in “Figure 1” as published: the dates on the axis were switched. The corrected figure and its caption appear below.

**Figure 1:**
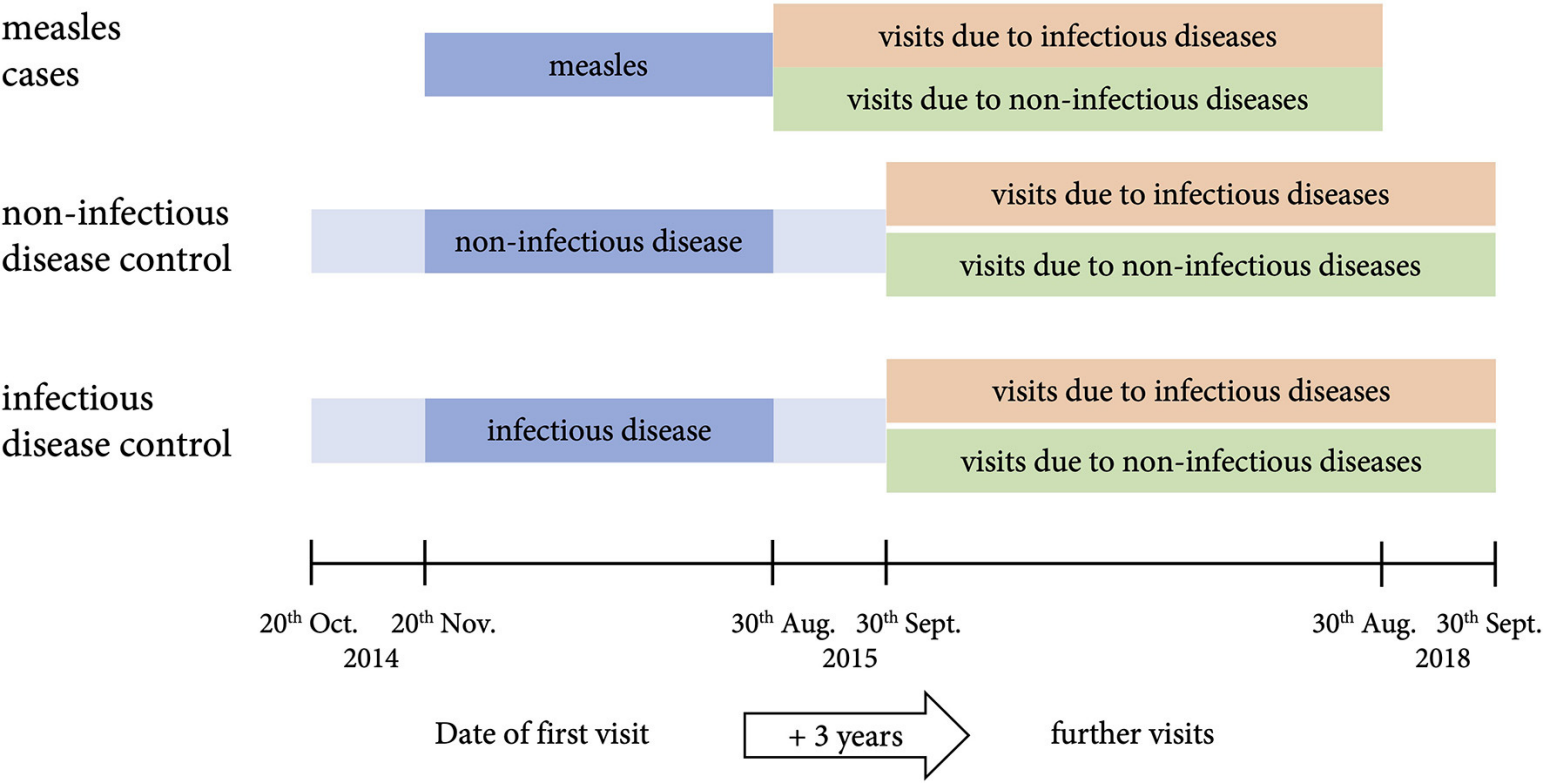
Study design. Children and adolescents (age ≤ 18 years) who fulfilled the WHO case definition for measles and presented to the emergency room during the measles epidemic between 20th October 2014 and 30th August 2015 were included as cases. Controls were matched for sex, age (± 10), date of first visit to the hospital (± 1 month, illustrated), and duration of in-patient care if applicable (± 20% in full days). All patients were followed up for 3 years.

The authors apologize for this error and state that this does not change the scientific conclusions of the article in any way. The original article has been updated.

## Publisher's note

All claims expressed in this article are solely those of the authors and do not necessarily represent those of their affiliated organizations, or those of the publisher, the editors and the reviewers. Any product that may be evaluated in this article, or claim that may be made by its manufacturer, is not guaranteed or endorsed by the publisher.

