# Supplementary material for: Specifically Increased Rate of Infections in Children Post Measles in a High Resource Setting
Source: Front Pediatr. 2022 Jun 9;10:896086. doi: 10.3389/fped.2022.896086 (PMC9261986; doi:10.3389/fped.2022.896086)
Supplement: Supplementary file 1 [file Data_Sheet_1.pdf]

Supplementary Material "Specifically increased rate of infections in children post measles in a high resource setting"

Table of Contents

eTable 1. categorization of ICD-Codes..2
eTable 2. Changes in matching controls to cases...17
eTable 3. repeat visits due to infectious diseases..22
eFigure 1. Distribution of studied measles cases over Berlin...27
eFigure 2. Empirical cumulative distribution of age..28
eFigure 3. Registered inhabitants per district..29
eFigure 4. Number of infectious repeated visits per patient in percent per group........................30

eTable 1. categorization of ICD-Codes

ICD-Code	Description	category	approximate duration of infection (days)	cluster
A00	Cholera	1	n/a*	12
A01.4	Paratyphoid fever, unspecified	1	n/a*	12
A02.0	Salmonella enteritis	1	7	1
A04.5	Campylobacter enteritis	1	7	1
A04.7	Enterocolitis due to Clostridium difficile	1	7	1
A08.0	Rotaviral enteritis	1	7	1
A08.1	Acute gastroenteropathy due to Norwalk agent	1	7	1
A08.2	Adenoviral enteritis	1	7	1
A08.3	Other viral enteritis	1	7	1
A08.4	Viral intestinal infection, unspecified	1	7	1
A09.0	Infectious gastroenteritis and colitis, unspecified	1	7	1
A09.9	Other gastroenteritis and colitis, unspecified	1	7	1
A37.0	Whooping cough due to Bordetella pertussis	1	21	6
A38	Scarlet fever	1	14	12
A41.52	Sepsis due to Pseudomonas	1	28	12
A46	Erysipelas	1	14	8
A49.0	Methicillin susceptible Staphylococcus aureus infection, unspecified site	1	14	5
A54.9	Gonococcal infection, unspecified	1	5	10
A60.0	Herpesviral infection of genital organs and urogenital system	1	14	2
A87.0	Enteroviral meningitis	1	7	12
A88.8	Other specified viral infections of central nervous system	1	14	12
B00.0	Eczema herpeticum	1	14	2
B00.2	Herpesviral gingivostomatitis and pharyngotonsillitis	1	14	2
B00.9	Herpesviral infection, unspecified	1	14	2
B01	Varicella	1	14	2
B02.2	Other postherpetic nervous system involvement	1	14	2
B05.1	Measles complicated by meningitis	1	21	13
B05.2	Measles complicated by pneumonia	1	21	13
B05.3	Measles complicated by otitis media	1	21	13
B05.4	Measles with intestinal complications	1	21	13
B05.8	Other measles complications	1	21	13
B05.9	Measles without complication	1	21	13
B08.2	Exanthema subitum [sixth disease], unspecified	1	5	2
B08.3	Erythema infectiosum [fifth disease]	1	14	11
B08.4	Enteroviral vesicular stomatitis with exanthem	1	7	7
B08.5	Enteroviral vesicular pharyngitis	1	7	5
B08.8	Other specified viral infections characterized by skin and mucous membrane lesions	1	7	11
B09	Unspecified viral infection characterized by skin and mucous membrane lesions	1	7	11
B27.0	Gammaherpesviral mononucleosis without complication	1	14	2

ICD-Code	Description	category	approximate duration of infection (days)	cluster
B34.0	Adenovirus infection, unspecified	1	7	11
B34.1	Enterovirus infection, unspecified	1	7	11
B34.8	Other viral infections of unspecified site	1	7	11
B34.9	Viral infection, unspecified	1	7	11
B37	Candidal stomatitis	1	14	8
B37.2	Candidiasis of skin and nail	1	14	8
B38	Coccidioidomycosis	1	n/a*	12
B80	Enterobiasis	1	3	1
B85.0	Pediculosis due to Pediculus humanus capitis	1	3	12
B86	Scabies	1	n/a*	12
B99	Other and unspecified infectious disease	1	7	11
C83.5	Lymphoblastic (diffuse) lymphoma	2	n/a	14
C91.0	Acute lymphoblastic leukemia	2	n/a	14
D16.9	Benign neoplasm of bone and articular cartilage, unspecified	2	n/a	14
D18.18	Lymphangioma, any site	2	n/a	14
D48.5	Neoplasm of uncertain behavior of skin	2	n/a	14
D69.0	Allergic purpura	2	n/a	14
D69.3	Immune thrombocytopenic purpura	2	n/a	14
D84.1	Defects in the complement system	2	n/a	14
E16.2	Hypoglycemia, unspecified	2	n/a	14
F41.0	Panic disorder [episodic paroxysmal anxiety]	2	n/a	14
F45.33	Somatoform disorder	2	n/a	14
F82.9	Specific developmental disorder of motor function	2	n/a	14
G00.1	Pneumococcal meningitis	1	14	12
G40.3	Generalized idiopathic epilepsy and epileptic syndromes	2	n/a	14
G41.0	Grand-Mal-Status	2	n/a	14
G43.0	Migraine without aura	2	n/a	14
G43.3	Complicated migraine	2	n/a	14
G43.8	Other migraine	2	n/a	14
G57.3	Lesion of lateral popliteal nerve	2	n/a	14
G58.0	Intercostal neuropathy	2	n/a	14
H00.0	Hordeolum	1	14	3
H02.8	Other specified disorders of eyelid	2	n/a	14
H05.0	Acute inflammation of orbit	1	21	3
H05.9	Unspecified disorder of orbit	2	n/a	14
H10	Conjungtivitis	1	5	3
H10.0	Mucopurulent conjunctivitis	1	5	3
H10.2	other acute conjungtivitis	1	5	3
H10.3	Unspecified acute conjunctivitis	1	5	3
H10.8	Other conjunctivitis	1	5	3
H10.9	Unspecified conjunctivitis	1	5	3

ICD-Code	Description	category	approximate duration of infection (days)	cluster
H11.3	Conjunctival hemorrhage	2	n/a	14
H11.9	Unspecified disorder of conjunctiva	2	n/a	14
H16.0	Corneal ulcer	1	14	3
H35.1	Retinopathy of prematurity	2	n/a	14
H50.0	Unspecified esotropia	2	n/a	14
H57.8	Other specified disorders of eye and adnexa	2	n/a	14
H60.1	Cellulitis of external ear	1	14	4
H60.3	Other infective otitis externa	1	7	4
H60.9	Unspecified otitis externa	1	7	4
H65	Acute nonsuppurative otitis media	1	3	4
H65.0	Acute serous otitis media	1	3	4
H65.1	Other acute nonsuppurative otitis media	1	3	4
H65.2	Chronic serous otitis media	1	3	4
H65.4	Other chronic nonsuppurative otitis media	1	3	4
H65.9	Unspecified nonsuppurative otitis media	1	3	4
H66.0	Acute suppurative otitis media	1	7	4
H66.4	Suppurative otitis media, unspecified	1	7	4
H66.9	Otitis media, unspecified	1	7	4
H67.1	Otitis media in diseases classified elsewhere	1	7	4
H70.0	Acute mastoiditis	1	14	4
H73.9	Unspecified disorder of tympanic membrane	2	n/a	14
H91.2	Sudden idiopathic hearing loss	2	n/a	14
H92.0	Otalgia	2	n/a	14
H93.2	Other abnormal auditory perceptions	2	n/a	14
I46.0	Cardiac arrest with successful resuscitation	2	n/a	14
I47.1	Supraventricular tachycardia	2	n/a	14
I80.28	Phlebitis and thrombophlebitis of other deep vessels of lower extremity	2	n/a	14
I88.0	Nonspecific mesenteric lymphadenitis	2	n/a	14
I88.1	Chronic lymphadenitis, except mesenteric	2	n/a	14
I88.8	Other nonspecific lymphadenitis	2	n/a	14
I88.9	Nonspecific lymphadenitis, unspecified	2	n/a	14
I89.09	Lymphedema, not elsewhere classified	2	n/a	14
I99	Other and unspecified disorder of circulatory system	2	n/a	14
J00	Acute nasopharyngitis [common cold]	1	10	11
J02	Acute pharyngitis	1	4	5
J02.0	Streptococcal pharyngitis	1	14	5
J02.8	Acute pharyngitis due to other specified organisms	1	4	5
J02.9	Acute pharyngitis, unspecified	1	4	5
J03	Acute tonsillitis	1	14	5
J03.0	Acute streptococcal tonsillitis, unspecified	1	14	5
J03.8	Acute tonsillitis due to other specified organisms	1	14	5

ICD-Code	Description	category	approximate duration of infection (days)	cluster
J03.9	Acute tonsillitis, unspecified	1	14	5
J04.0	Acute laryngitis	1	4	5
J04.2	Acute laryngotracheitis	1	4	5
J05.0	Acute obstructive laryngitis [croup]	1	2	5
J06.0	Acute laryngopharyngitis	1	2	5
J06.8	Acute upper respiratory infections of multiple and unspecifies sites	1	10	11
J06.9	Acute upper respiratory infection, unspecified	1	10	11
J09	Influenza due to certain identified influenza viruses	1	10	5
J10.0	Influenza due to seasonal influenza virus with pneumonia	1	10	6
J11.1	Influenza due to unidentified influenza virus with other respiratory manifestations	1	10	5
J12.1	Respiratory syncytial virus pneumonia	1	14	6
J12.8	Other viral pneumonia	1	14	6
J13	Pneumonia due to Streptococcus pneumoniae	1	14	6
J15.7	Pneumonia due to Mycoplasma pneumoniae	1	14	6
J15.8	Pneumonia due to other specified bacteria	1	14	6
J15.9	Unspecified bacterial pneumonia	1	14	6
J17.1	Pneumonia in diseases classified elsewhere	1	14	6
J18.0	Bronchopneumonia, unspecified organism	1	14	6
J18.1	Lobar pneumonia, unspecified organism	1	14	6
J18.8	Other pneumonia, unspecified organism	1	14	6
J18.9	Pneumonia, unspecified organism	1	14	6
J20	Acute bronchitis	1	7	6
J20.5	Acute bronchitis due to respiratory syncytial virus	1	7	6
J20.8	Acute bronchitis due to other specified organisms	1	7	6
J20.9	Acute bronchitis, unspecified	1	7	6
J21.0	Acute bronchiolitis due to respiratory syncytial virus	1	21	6
J22	Unspecified acute lower respiratory infection	1	14	6
J35.0	Chronic tonsillitis	1	14	5
J35.1	Hypertrophy of tonsils	2	n/a	14
J35.2	Hypertrophy of adenoids	2	n/a	14
J35.3	Hypertrophy of tonsils with hypertrophy of adenoids	2	n/a	14
J35.8	Other chronic diseases of tonsils and adenoids	2	n/a	14
J38.5	Laryngeal spasm	1	2	5
J40	Bronchitis, not specified as acute or chronic	1	7	6
J41.1	Mucopurulent chronic bronchitis	1	7	6
J44.89	Chronic obstructive pulmonary disease, unspecified	1	14	6
J45.9	Unspecified asthma	2	n/a	14
J98.8	Other specified respiratory disorders	2	n/a	14
K00.7	Teething syndrome	2	n/a	14
K01.0	Embedded teeth	2	n/a	14
K02.9	Dental caries, unspecified	1	n/a*	7

ICD-Code	Description	category	approximate duration of infection (days)	cluster
K04.7	Periapical abscess without sinus	1	n/a*	7
K05.0	Acute gingivitis	1	n/a*	7
K08.1	Complete loss of teeth due to trauma, extraction or localised peridontal diseases	2	n/a	14
K08.88	Other specified disorders of teeth and supporting structures	2	n/a	14
K08.9	Disorder of teeth and supporting structures, unspecified	2	n/a	14
K11.2	Sialoadenitis	1	n/a*	7
K12.0	Recurrent oral aphthae	1	7	7
K12.1	Other forms of stomatitis	1	7	7
K12.28	Cellulitis and abscess of mouth	1	14	7
K20	Esophagitis	1	n/a*	1
K21.0	Gastro-esophageal reflux disease with esophagitis	2	n/a	14
K21.9	Gastro-esophageal reflux disease without esophagitis	2	n/a	14
K29.1	Other acute gastritis	1	7	1
K29.5	Unspecified chronic gastritis	2	n/a	14
K29.7	Gastritis, unspecified	1	7	1
K35	Acute appendicitis	1	n/a*	12
K35.2	Acute appendicitis with generalized peritonitis	1	n/a*	12
K35.30	Acute appendicitis with localized peritonitis, without perforation or gangrene	1	n/a*	12
K35.32	Acute appendicitis with abscess	1	n/a*	12
K35.8	Unspecified acute appendicitis	1	n/a*	12
K36	Other appendicitis	1	n/a*	12
K37	Unspecified appendicitis	1	n/a*	12
K40.20	Bilateral inguinal hernia, without obstruction or gangrene, not specified as recurrent	2	n/a	14
K40.30	Unilateral inguinal hernia, with obstruction, without gangrene, not specified as recurrent	2	n/a	14
K40.31	Unilateral inguinal hernia, with obstruction, without gangrene, recurrent	2	n/a	14
K40.9	Unilateral inguinal hernia, without obstruction or gangrene	2	n/a	14
K40.90	Unilateral inguinal hernia, without obstruction or gangrene, not specified as recurrent	2	n/a	14
K42.9	Umbilical hernia without obstruction or gangrene	2	n/a	14
K44.9	Diaphragmatic hernia without obstruction or gangrene	2	n/a	14
K52.1	Toxic gastroenteritis and colitis	2	n/a	14
K52.9	Noninfective gastroenteritis and colitis, unspecified	2	n/a	14
K56.1	Intussusception	2	n/a	14
K56.7	Ileus, unspecified	2	n/a	14
K59.0	Constipation	2	n/a	14
K59.9	Functional intestinal disorder, unspecified	2	n/a	14
K60.2	Anal fissure, unspecified	2	n/a	14
K61.0	Anal abscess	2	n/a	14
K64.8	Other hemorrhoids	2	n/a	14
K65.0	Generalized (acute) peritonitis	1	n/a*	12
K66.0	Peritoneal adhesions (postprocedural) (postinfection)	2	n/a	14

ICD-Code	Description	category	approximate duration of infection (days)	cluster
K83.1	Obstruction of bile duct	2	n/a	14
K86.8	Other specified diseases of pancreas	2	n/a	14
K92.1	Melena	2	n/a	14
L01.0	Impetigo, unspecified	1	5	8
L02.2	Cutaneous abscess, furuncle and carbuncle of trunk	1	5	8
L02.4	Cutaneous abscess, furuncle and carbuncle of limb	1	5	8
L02.8	Cutaneous abscess, furuncle and carbuncle of other sites	1	5	8
L03.01	Cellulitis and acute lymphangitis of finger	1	14	8
L03.02	Cellulitis and acute lymphangitis of toe	1	14	8
L03.10	Cellulitis of upper limb	1	14	8
L03.11	Cellulitis of lower limb	1	14	8
L03.2	Cellulitis of face	1	14	8
L04.0	Acute lymphadenitis of face, head and neck	1	7	8
L05.0	Pilonidal cyst with abscess	1	14	8
L08.9	Local infection of the skin and subcutaneous tissue, unspecified	1	7	8
L20.8	Other seborrheic dermatitis	2	n/a	14
L20.9	Seborrheic dermatitis, unspecified	2	n/a	14
L22	Diaper dermatitis	1	14	8
L23.9	Allergic contact dermatitis, unspecified cause	2	n/a	14
L28.2	Other prurigo	2	n/a	14
L30.9	Dermatitis, unspecified	2	n/a	14
L44.4	Infantile papular acrodermatitis [Gianotti-Crosti]	2	n/a	14
L50.0	Allergic urticaria	2	n/a	14
L50.1	Idiopathic urticaria	2	n/a	14
L50.6	Contact urticaria	2	n/a	14
L50.8	Other urticaria	2	n/a	14
L50.9	Urticaria, unspecified	2	n/a	14
L51	Nonbullous erythema multiforme	2	n/a	14
L51.9	Erythema multiforme, unspecified	2	n/a	14
L53.8	Other specified erythematous conditions	2	n/a	14
L56.2	Photocontact dermatitis [berloque dermatitis]	2	n/a	14
L73.9	Follicular disorder, unspecified	2	n/a	14
L85.3	Xerosis cutis	2	n/a	14
M00.99	Pyogenic arthritis, unspecified	1	14	9
M02.96	Reactive arthropathy, unspecified, lower limb	2	n/a	14
M12.8	Other specific arthropathies, not elsewhere classified	2	n/a	14
M12.80	Other specific arthropathies, not elsewhere classified, multiple sites	2	n/a	14
M12.85	Other specific arthropathies, not elsewhere classified, unspecified hip	2	n/a	14
M23.99	Unspecified internal derangement of unspecified knee	2	n/a	14
M25.46	Effusion, lower limb	2	n/a	14
M25.47	Effusion, ancle and foot	2	n/a	14

ICD-Code	Description	category	approximate duration of infection (days)	cluster
M25.51	Pain in unspecified shoulder	2	n/a	14
M25.52	Pain in unspecified elbow	2	n/a	14
M25.53	Pain in unspecified wrist	2	n/a	14
M25.56	Pain in unspecified knee	2	n/a	14
M25.75	Osteophyte, unspecified hip	2	n/a	14
M41.15	Juvenile idiopathic scoliosis, thoracolumbar region	2	n/a	14
M43.6	Torticollis	2	n/a	14
M54.2	Cervicalgia	2	n/a	14
M54.4	Lumbago with sciatica, unspecified side	2	n/a	14
M54.84	Other dorsalgia, thoracic region	2	n/a	14
M54.96	Dorsalgia, unspecified, lumbar region	2	n/a	14
M62.88	Other specified disorders of muscle, other region	2	n/a	14
M62.89	Other specified disorders of muscle, region not specified	2	n/a	14
M65.3	Trigger finger	2	n/a	14
M77.5	Other enthesopathy of unspecified foot and ankle	2	n/a	14
M79.58	Residual foreign body in soft tissue, other region	2	n/a	14
M79.60	Pain in multiple limbs	2	n/a	14
M79.64	Pain in unspecified hand	2	n/a	14
M79.69	Pain in unspecified limb	2	n/a	14
M86.07	Acute hematogenous osteomyelitis, unspecified ankle and foot	1	14	9
N10	Acute pyelonephritis	2	n/a	14
N28.8	Other specified disorders of kidney and ureter	2	n/a	14
N30.0	Acute cystitis	1	7	10
N30.9	Cystitis, unspecified	1	7	10
N39.0	Urinary tract infection, site not specified	1	7	10
N43.2	Other hydrocele	2	n/a	14
N43.3	Hydrocele, unspecified	2	n/a	14
N44.0	Torsion of testis, unspecified	2	n/a	14
N44.1	Torsion of testis, unspecified	2	n/a	14
N45.9	Orchitis, epididymitis and epididymoorchitis without abscess	1	14	10
N47	Redundant prepuce, phimosis and paraphimosis	2	n/a	14
N48.1	Balanitis	1	5	10
N48.8	Other specified disorders of penis	2	n/a	14
N50.8	Other specified disorders of the male genital organs	2	n/a	14
N51.2	Balanitis in diseases classified elsewhere	1	5	10
N61	Inflammatory disorders of breast	1	n/a*	8
N70.0	Acute salpingitis and oophoritis	1	n/a*	10
N70.9	Salpingitis and oophoritis, unspecified	1	14	10
N76.0	Acute vaginitis	1	14	10
N76.2	Acute vulvitis	1	5	10
N76.88	Other specified inflammation of vagina and vulva	1	5	10

ICD-Code	Description	category	approximate duration of infection (days)	cluster
N83.2	Unspecified ovarian cyst, unspecified side	2	n/a	14
N92.1	Excessive and frequent menstruation with irregular cycle	2	n/a	14
N92.5	Other specified irregular menstruation	2	n/a	14
N94.4	Primary dysmenorrhea	2	n/a	14
N94.9	Unspecified condition associated with female genital organs and menstrual cycle	2	n/a	14
O06.9	Unspecified abortion	2	n/a	14
O23.0	Infections of kidney in pregnancy	1	5	10
O41.0	Oligohydramnios	2	n/a	14
O60.0	Preterm labor without delivery	2	n/a	14
O60.3	Preterm delivery without spontaneous labour	2	n/a	14
O70.1	Second degree perineal laceration during delivery	2	n/a	14
P78.8	Other specified perinatal digestive system disorders	2	n/a	14
Q12.0	Congenital cataract	2	n/a	14
Q18.0	Sinus, fistula and cyst of branchial cleft	2	n/a	14
Q53.1	Unspecified undescended testicle, unilateral	2	n/a	14
Q53.2	Undescended testicle, unspecified, bilateral	2	n/a	14
Q54.0	Hypospadias, balanic	2	n/a	14
Q55.2	Unspecified congenital malformations of testis and scrotum	2	n/a	14
Q66.0	Congenital talipes equinovarus	2	n/a	14
R00.0	Tachycardia, unspecified	2	n/a	14
R00.2	Palpitations	2	n/a	14
R04.0	Epistaxis	2	n/a	14
R05	Cough	2	n/a	14
R06.0	Dyspnea, unspecified	2	n/a	14
R06.4	Hyperventilation	2	n/a	14
R07.0	Pain in throat	2	n/a	14
R07.1	Chest pain on breathing	2	n/a	14
R07.3	Other chest pain	2	n/a	14
R07.4	Chest pain, unspecified	2	n/a	14
R10.0	Acute abdomen	2	n/a	14
R10.1	Upper abdominal pain, unspecified	2	n/a	14
R10.3	Lower abdominal pain, unspecified	2	n/a	14
R10.4	Unspecified abdominal pain	2	n/a	14
R11	Nausea and vomiting	2	n/a	14
R13.9	Other dysphagia	2	n/a	14
R14	Abdominal distension (gaseous)	2	n/a	14
R19.80	Abdominal compartment sydrome	2	n/a	14
R19.88	Other specified symptoms and signs involving the digestive system and abdomen	2	n/a	14
R20.2	Paresthesia of skin	2	n/a	14
R20.3	Hyperesthesia	2	n/a	14
R20.8	Other disturbances of skin sensation	2	n/a	14

ICD-Code	Description	category	approximate duration of infection (days)	cluster
R21	Rash and other nonspecific skin eruption	2	n/a	14
R22.0	Localized swelling, mass and lump, head	2	n/a	14
R23.0	Cyanosis	2	n/a	14
R23.3	Spontaneous ecchymoses	2	n/a	14
R26.8	Other abnormalities of gait and mobility	2	n/a	14
R30.0	Dysuria	2	n/a	14
R40.0	Somnolence	2	n/a	14
R42	Dizziness and giddiness	2	n/a	14
R45.1	Restlessness and agitation	2	n/a	14
R50.80	Fever of unknown origin	1	7	11
R50.9	Fever, unspecified	1	7	11
R51	Headache	2	n/a	14
R52.9	Pain, unspecified	2	n/a	14
R55	Syncope and collapse	2	n/a	14
R56.0	Febrile convulsions	1	4	11
R56.8	Unspecified convulsions	2	n/a	14
R59.0	Localized enlarged lymph nodes	2	n/a	14
R62.8	Other lack of expected normal physiological development in childhood	2	n/a	14
R78.8	Finding of other specified substances, not normally found in blood	2	n/a	14
S00.05	Superficial injury of scalp	2	n/a	14
S00.08	Superficial injury of scalp, other	2	n/a	14
S00.1	Contusion of unspecified eyelid and periocular area	2	n/a	14
S00.23	Other superficial injuries of eyelid and periocular area	2	n/a	14
S00.35	Superficial injury of nose	2	n/a	14
S00.45	Superficial injury of ear	2	n/a	14
S00.50	Superficial injury of lip and oral cavity, unspecified	2	n/a	14
S00.58	Superficial injury of lip and oral cavity, other	2	n/a	14
S00.81	Abrasion of other part of head	2	n/a	14
S00.85	Superficial injury of other parts of head	2	n/a	14
S00.9	Unspecified superficial injury of unspecified part of head	2	n/a	14
S00.95	Unspecified superficial injury of unspecified part of head, bruise	2	n/a	14
S00.98	Unspecified superficial injury of unspecified part of head, other	2	n/a	14
S01.0	Unspecified open wound of scalp	2	n/a	14
S01.1	Unspecified open wound of eyelid and periocular area	2	n/a	14
S01.20	Unspecified open wound of eyelid and periocular area, initial encounter	2	n/a	14
S01.21	Laceration without foreign body of nose	2	n/a	14
S01.30	Unspecified open wound of ear	2	n/a	14
S01.31	Laceration without foreign body of ear	2	n/a	14
S01.41	Laceration without foreign body of cheek and temporomandibular area	2	n/a	14
S01.49	Laceration without foreign body of cheek and temporomandibular area, other and multiple regions	2	n/a	14
S01.51	Unspecified open wound of lip	2	n/a	14

ICD-Code	Description	category	approximate duration of infection (days)	cluster
S01.54	Unspecified open wound of tounge	2	n/a	14
S01.55	Unspecified open wound of oral cavity	2	n/a	14
S01.59	Unspecified open wound of oral cavity, other and multiple regions	2	n/a	14
S01.80	Unspecified open wound of other part of head	2	n/a	14
S01.9	Unspecified open wound of unspecified part of head	2	n/a	14
S02.0	Fracture of vault of skull	2	n/a	14
S02.1	Fracture of base of skull	2	n/a	14
S02.2	Fracture of nasal bones	2	n/a	14
S02.8	Fracture of other specified skull and facial bones	2	n/a	14
S02.9	Unspecified fracture of skull	2	n/a	14
S03.2	Dislocation of tooth	2	n/a	14
S05.0	Injury of conjunctiva and corneal abrasion without foreign body	2	n/a	14
S05.1	Contusion of eyeball and orbital tissues	2	n/a	14
S06.0	Concussion without loss of consciousness	2	n/a	14
S06.9	Unspecified intracranial injury without loss of consciousness	2	n/a	14
S09.9	Unspecified injury of head	2	n/a	14
S10.83	Contusion of other specified part of neck	2	n/a	14
S10.85	Superficial foreign body of other specified part of neck, initial encounter	2	n/a	14
S13.4	Sprain of ligaments of cervical spine	2	n/a	14
S13.6	Sprain of joints and ligaments of other parts of neck	2	n/a	14
S20.2	Contusion of thorax	2	n/a	14
S27.31	Unspecified injury of lung	2	n/a	14
S30.0	Contusion of lower back and pelvis	2	n/a	14
S30.1	Contusion of abdominal wall	2	n/a	14
S30.2	Contusion of unspecified external genital organ	2	n/a	14
S30.81	Abrasion of abdominal wall	2	n/a	14
S30.95	Unspecified superficial injury of lower back and pelvis	2	n/a	14
S31.0	Unspecified open wound of lower back and pelvis	2	n/a	14
S39.9	Unspecified injury of abdomen, lower back or pelvis	2	n/a	14
S40.0	Contusion of shoulder and upper arm	2	n/a	14
S41.0	Unspecified open wound of shoulder	2	n/a	14
S41.1	Unspecified open wound of upper arm	2	n/a	14
S42.00	Fracture of unspecified part of clavicle	2	n/a	14
S42.01	Fracture of sternal end of clavicle	2	n/a	14
S42.02	Fracture of shaft of clavicle	2	n/a	14
S42.20	Unspecified fracture of upper end of humerus	2	n/a	14
S42.22	Fracture of surgical neck of humerus	2	n/a	14
S42.23	Fracture of anatomical neck of humerus	2	n/a	14
S42.3	Fracture of shaft of humerus	2	n/a	14
S42.41	Supracondylar fracture of humerus	2	n/a	14
S42.43	Fracture of medial epicondyle of humerus	2	n/a	14

ICD-Code	Description	category	approximate duration of infection (days)	cluster
S42.45	Transcondylar fracture of humerus	2	n/a	14
S42.49	Other fracture of lower end of humerus	2	n/a	14
S43.4	Unspecified sprain of shoulder joint	2	n/a	14
S50.0	Contusion of elbow	2	n/a	14
S50.1	Contusion of forearm, unspecified	2	n/a	14
S51.9	Unspecified open wound of forearm	2	n/a	14
S52.0	Fracture of upper end of ulna	2	n/a	14
S52.00	Fracture of upper end of ulna, unspecified	2	n/a	14
S52.11	Fracture of head of radius	2	n/a	14
S52.12	Fracture of neck of radius	2	n/a	14
S52.30	Fracture of shaft of radius	2	n/a	14
S52.4	Fracture of shaft of ulna and radius, combined	2	n/a	14
S52.50	Fracture of the lower end of radius, unspecified	2	n/a	14
S52.6	Fracture of the lower end of ulna, unspecified	2	n/a	14
S52.8	Unspecified other fracture of forearm	2	n/a	14
S53.0	Subluxation of radial head	2	n/a	14
S53.40	Sprain of elbow, unspecified	2	n/a	14
S53.48	Other sprain of elbow	2	n/a	14
S60.0	Contusion of unspecified finger without damage to nail	2	n/a	14
S60.2	Contusion of unspecified wrist	2	n/a	14
S60.81	Abrasion of unspecified wrist	2	n/a	14
S60.84	Superficial foreign body of unspecified wrist	2	n/a	14
S61.0	Open wound of finger without damage to nail	2	n/a	14
S61.1	Open wound of finger with damage to nail	2	n/a	14
S61.9	Open wound of wrist, unspecified	2	n/a	14
S62.0	Fracture of navicular [scaphoid] bone of wrist	2	n/a	14
S62.21	Fracture of first metacarpal bone, base	2	n/a	14
S62.3	Fracture of unspecified metacarpal bone	2	n/a	14
S62.30	Fracture of unspecified metacarpal bone, unspecified	2	n/a	14
S62.60	Fracture of other finger, unspecified	2	n/a	14
S62.61	Fracture of proximal phalanx of other finger	2	n/a	14
S62.62	Fracture of middle phalanx of other finger	2	n/a	14
S62.63	Fracture of distal phalanx of other finger	2	n/a	14
S63.52	Sprain of radiocarpal joint of wrist	2	n/a	14
S63.6	Sprain of unspecified finger	2	n/a	14
S63.60	Sprain of unspecified finger, unspecified	2	n/a	14
S63.68	Sprain of unspecified finger, other	2	n/a	14
S66.2	Laceration of extensor muscle, fascia and tendon of thumb at wrist and hand level	2	n/a	14
S68.1	Complete or partial traumatic amputation of other finger	2	n/a	14
S70.0	Contusion of hip	2	n/a	14
S70.1	Contusion of thigh	2	n/a	14

ICD-Code	Description	category	approximate duration of infection (days)	cluster
S71.0	Open wound of hip	2	n/a	14
S72.3	Fracture of shaft of femur	2	n/a	14
S76.4	Unspecified injury of other specified muscles, fascia and tendons at thigh level	2	n/a	14
S80.0	Contusion of knee	2	n/a	14
S80.1	Contusion of lower leg, unspecified	2	n/a	14
S80.81	Abrasion of lower leg	2	n/a	14
S81.0	Open wound of knee	2	n/a	14
S81.9	Open wound of lower leg, unspecified	2	n/a	14
S82.18	Other fracture of upper end of tibia	2	n/a	14
S82.2	Fracture of shaft of tibia	2	n/a	14
S82.21	Fracture of shaft of tibia with fracture of fibula	2	n/a	14
S82.28	Other fracture of shaft of tibia	2	n/a	14
S82.3	Fracture of lower end of tibia	2	n/a	14
S82.31	Fracture of lower end of tibia with fracture of fibula	2	n/a	14
S82.38	Other fracture of lower end of tibia	2	n/a	14
S82.40	Fracture of fibula, unspecified	2	n/a	14
S82.6	Fracture of lateral malleolus of fibula	2	n/a	14
S83.0	Subluxation of patella	2	n/a	14
S83.10	Subluxation of knee, unspecified	2	n/a	14
S83.50	Sprain of unspecified cruciate ligament of knee	2	n/a	14
S83.53	Sprain of anterior cruciate ligament of knee	2	n/a	14
S83.6	Sprain of other specified parts of knee	2	n/a	14
S86.1	Unspecified injury of other muscle(s) and tendon(s) of posterior muscle group at lower leg level	2	n/a	14
S90.0	Contusion of ankle	2	n/a	14
S90.1	Contusion of lesser toe(s) without damage to nail	2	n/a	14
S90.3	Contusion of unspecified parts of foot	2	n/a	14
S90.7	Unspecified superficial injury of ankle and foot	2	n/a	14
S90.81	Abrasion of ankle and foot	2	n/a	14
S90.82	Blister (nonthermal) of ankle and foot	2	n/a	14
S90.83	Insect bite (nonvenomous) of ankle and foot	2	n/a	14
S90.84	Superficial foreign body of ankle and foot	2	n/a	14
S91.1	Open wound of lesser toe(s) without damage to nail	2	n/a	14
S91.2	Open wound of lesser toe(s) with damage to nail	2	n/a	14
S91.3	Open wound of unspecified parts of foot	2	n/a	14
S92.2	Fracture of tarsal bone(s) of foot	2	n/a	14
S92.3	Fracture of metatarsal bone(s) of foot	2	n/a	14
S92.4	Fracture of great toe	2	n/a	14
S92.5	Fracture of lesser toe(s)	2	n/a	14
S93.2	Rupture of ligaments at ankle and foot level	2	n/a	14
S93.4	Sprain of ankle	2	n/a	14
S93.40	Sprain of ankle, unspecified	2	n/a	14

ICD-Code	Description	category	approximate duration of infection (days)	cluster
S93.43	Sprain of tibiofibular ligament of ankle	2	n/a	14
S93.48	Sprain of other ligament of ankle	2	n/a	14
S93.5	Sprain of metatarsophalangeal joint of toe(s)	2	n/a	14
S93.6	Sprain and strain of other and unspecified parts of foot	2	n/a	14
S96.9	Unspecified injury of unspecified muscles and tendons at ankle and foot level, right foot, subsequent encounter	2	n/a	14
S99.9	Unspecified injury of ankle	2	n/a	14
T00.0	Superficial injuries involving head with neck	2	n/a	14
T09.05	Contusion of unspecified muscle and tendon of trunk	2	n/a	14
T10.0	Fracture of upper limb, level unspecified	2	n/a	14
T11.05	Contusion of upper limb, level unspecified	2	n/a	14
T11.9	Unspecified injury of upper limb, level unspecified	2	n/a	14
T13.05	Contusion of lower limb, level unspecified	2	n/a	14
T13.9	Unspecified injury of lower limb, level unspecified	2	n/a	14
T14.01	Abrasion of unspecified body region	2	n/a	14
T14.03	Insect bite (nonvenomous) of unspecified body region	2	n/a	14
T14.05	Contusion of unspecified body region	2	n/a	14
T14.1	Open wound of unspecified body region	2	n/a	14
T14.6	Injury of muscles and tendons of unspecified body region	2	n/a	14
T14.9	Injury, unspecified	2	n/a	14
T15.8	Foreign body in other and multiple parts of external eye	2	n/a	14
T16	Foreign body in ear	2	n/a	14
T17.1	Foreign body in nostril	2	n/a	14
T17.2	Foreign body in pharynx	2	n/a	14
T17.4	Foreign body in trachea	2	n/a	14
T17.9	Foreign body in respiratory tract, part unspecified	2	n/a	14
T18.0	Foreign body in mouth	2	n/a	14
T18.1	Foreign body in esophagus	2	n/a	14
T18.2	Foreign body in stomach	2	n/a	14
T18.8	Foreign body in other parts of alimentary tract	2	n/a	14
T18.9	Foreign body of alimentary tract, part unspecified	2	n/a	14
T20.0	Burn of unspecified degree of head, face, and neck	2	n/a	14
T20.20	Burn of second degree of head, face, and neck	2	n/a	14
T21.00	Burn of unspecified degree of trunk	2	n/a	14
T21.15	Burn of first degree of genital region	2	n/a	14
T21.23	Burn of second degree of abdominal wall	2	n/a	14
T22.10	Burn of first degree of shoulder and upper limb, except wrist and hand	2	n/a	14
T22.2	Burn of second degree of shoulder and upper limb, except wrist and hand	2	n/a	14
T22.21	Burn of second degree of forearm and elbow	2	n/a	14
T22.22	Burn of second degree of upper arm and shoulder	2	n/a	14
T23.0	Burn of unspecified degree of hand	2	n/a	14
T23.20	Burn of second degree of hand	2	n/a	14

ICD-Code	Description	category	approximate duration of infection (days)	cluster
T23.3	Burn of third degree of hand	2	n/a	14
T24.0	Burn of unspecified degree of lower limb, except ankle and foot	2	n/a	14
T24.20	Burn of second degree of lower limb, except ankle and foot	2	n/a	14
T24.21	Burn of second degree of lower limb, except ankle and foot	2	n/a	14
T25.0	Burn of unspecified degree of ankle and foot	2	n/a	14
T25.20	Burn of second degree of ankle and foot	2	n/a	14
T29.20	Burns of multiple regions, no more than second-degree burns mentioned	2	n/a	14
T30.0	Burn of unspecified body region, unspecified degree	2	n/a	14
T30.1	Burn of first degree, body region unspecified	2	n/a	14
T30.20	Burn of second degree, body region unspecified	2	n/a	14
T39.1	Poisoning by 4-Aminophenol derivatives	2	n/a	14
T46.5	Poisoning by other antihypertensive drugs	2	n/a	14
T50.8	Poisoning by diagnostic agents	2	n/a	14
T50.9	Other and unspecified drugs, medicaments and biological substances	2	n/a	14
T51.9	Toxic effect of unspecified alcohol	2	n/a	14
T55	Toxic effect of soaps	2	n/a	14
T60.9	Toxic effect of unspecified pesticide	2	n/a	14
T62.1	Toxic effect of ingested berries	2	n/a	14
T62.9	Toxic effect of unspecified noxious substance eaten as food	2	n/a	14
T63.4	Toxic effect of venom of centipedes and venomous millipedes	2	n/a	14
T63.8	Toxic effect of contact with other venomous animals	2	n/a	14
T65.9	Toxic effect of unspecified substance	2	n/a	14
T67.0	Heatstroke and sunstroke	2	n/a	14
T70.8	Other effects of air pressure and water pressure	2	n/a	14
T74.9	Unspecified adult maltreatment	2	n/a	14
T75.1	Unspecified effects of drowning and nonfatal submersion	2	n/a	14
T75.4	Electrocution	2	n/a	14
T78.0	Anaphylactic reaction due to unspecified food	2	n/a	14
T78.1	Other adverse food reactions, not elsewhere classified	2	n/a	14
T78.4	Allergy, unspecified	2	n/a	14
T79.3	Post-traumatic wound infection, not elsewhere classified	1	14	8
T79.9	Unspecified early complication of trauma	2	n/a	14
T81.0	Haemorrhage and haematoma complicating a procedure, not elsewhere classified	2	n/a	14
T81.9	Unspecified complication of procedure	2	n/a	14
T85.5	Mechanical complication of gastrointestinal prosthetic devices, implants and grafts	2	n/a	14
T88.1	Other complications following immunization, not elsewhere classified	2	n/a	14
Z01.5	Diagnostic skin and sensitization tests	2	n/a	14
Z03.3	Observation for suspected nervous system disorder	2	n/a	14
Z03.6	Observation for suspected toxic effect from ingested substance	2	n/a	14
Z03.8	Observation for other suspected diseases and conditions	2	n/a	14
Z03.9	Observation for suspected disease or condition, unspecified	2	n/a	14

ICD-Code	Description	category	approximate duration of infection (days)	cluster
Z20.8	Contact with and exposure to other communicable diseases	2	n/a	14
Z47.0	Follow-up care involving removal of fracture plate and other internal fixation device	2	n/a	14
Z71	Persons encountering health services for other counselling and medical advice, not elsewhere classified	2	n/a	14
Z76.9	Person encountering health services in unspecified circumstances	2	n/a	14
Z91.0	Personal history of allergy, other than to drugs and biological substances	2	n/a	14

Documented ICD10-Codes with description and assigned categories (infectious = 1, non-infectious = 2), which defined control groups and categorization of repeated visits.
Approximate duration of infection and clusters of body regions were used to define related visits due to an infection.
n/a: not applicable because ICD is non-infectious
n/a*: not applicable because unlikely to present again with the same disease
clusters: 1 = gastrointestinal, 2 = herpes, 3 = eye, 4 = ear, 5 = pharynx/larynx, 6 = lung, 7 = mouth, 8 = skin, 9 = joints/bones, 10 = urinary tract/kidney/genitalia, 11 = unspecified viridae, 12 = other, unrelated, 13 = measles, 14 = non-infectious

eTable 2. Changes in matching controls to cases

ID	sex	days difference from case date of presentation	case duration of stay	control duration of stay
005.2	-	34	-	-
006.1	male	46	-	-
007.1	male	42	-	-
007.4	male	32	-	-
009.1	-	39	4	2
009.2	-	-	4	2
009.4	male	45	-	-
010.1	-	46	-	-
014.1	male	36	-	-
014.3	-	43	-	-
026.2	male	-	-	-
030.2	-	31	-	-
031.1	female	-	-	-
040.1	male	-	-	-
040.2	male	-	-	-
040.3	male	-	-	-
040.4	male	-	-	-
049.1	male	41	11	4
049.2	male	-	11	2
049.3	-	31	-	-
049.4	male	-	11	6
051.2	-	38	-	-
051.3	-	47	-	-
051.4	female	33	-	-
053.1	female	-	-	-
053.2	female	-	-	-
053.3	female	-	-	-
053.4	female	-	-	-
062.2	female	-	-	-
067.1	-	40	-	-
067.2	-	46	-	-
067.3	female	44	-	-
071.2	male	-	-	-
076.2	female	38	-	-
076.3	-	33	-	-
087.2	female	31	-	-
089.2	male	-	-	-
097.1	-	36	-	-
097.4	-	41	-	-
098.2	-	41	-	-
099.1	male	-	-	-
099.3	-	36	-	-

ID	sex	days difference from case date of presentation	case duration of stay	control duration of stay
100.1	-	37	-	-
100.2	female	-	-	-
100.3	-	38	-	-
101.2	female	-	-	-
101.3	-	35	-	-
101.4	female	-	-	-
102.2	-	62	-	-
102.3	-	36	-	-
103.1	male	-	-	-
103.2	male	-	-	-
104.1	-	32	-	-
106.3	male	-	-	-
106.4	male	-	-	-
107.3	-	38	-	-
107.4	-	44	-	-
109.1	-	35	-	-
109.2	-	33	-	-
110.3	male	-	-	-
110.4	male	-	-	-
112.2	-	46	-	-
112.4	-	41	-	-
113.1	male	-	-	-
113.2	male	-	-	-
113.3	-	40	-	-
113.4	-	48	-	-
115.1	-	43	-	-
115.2	female	43	-	-
115.4	-	31	-	-
116.1	male	39	-	-
117.1	male	38	-	-
117.2	-	48	-	-
118.1	male	-	-	-
118.2	male	-	-	-
121.1	female	-	-	-
121.2	female	41	-	-
122.1	male	35	-	-
122.2	male	-	9	4
127.1	-	-	7	2
127.2	-	33	7	2
127.3	male	-	-	-
127.4	male	-	-	-
128.2	-	34	-	-
132.1	male	-	-	-
132.2	male	46	-	-

ID	sex	days difference from case date of presentation	case duration of stay	control duration of stay
132.4	-	32	-	-
133.2	-	33	-	-
134.1	-	45	-	-
134.2	-	-	8	3
134.4	-	48	-	-
135.1	-	37	-	-
135.3	male	-	-	-
136.1	male	-	-	-
136.2	male	-	7	3
136.4	male	40	-	-
138.4	-		-	-
143.3	-	31	-	-
149.1	-	31	-	-
172.2	female	-	-	-
173.3	male	-	-	-
173.4	male	-	-	-
176.1	female	38	-	-
176.2	-	-	3	1
176.4	female	40	-	-
180.1	female	41	-	-
180.2	female	-	-	-
180.3	female	31	-	-
186.2	-	32	-	-
187.3	-	32	-	-
187.4	male	-	-	-
189.2	male	-	-	-
189.3	-	39	-	-
190.1	-	38	-	-
191.1	-	41	4	2
191.2	-	-	4	2
191.3	-	37	-	-
191.4	-	33	-	-
193.1	female	-	-	-
193.2	female	-	-	-
193.3	-	45	-	-
193.4	female	-	-	-
198.2	-	43	-	-
198.3	male	-	-	-
198.4	male	38	-	-
199.1	female	-	-	-
199.2	female	-	-	-
200.1	male	32	-	-
201.4	female	-	-	-
202.2	-	42	-	-

ID	sex	days difference from case date of presentation	case duration of stay	control duration of stay
204.3	female	-	-	-
212.1	-	31	-	-
213.3	female	-	-	-
215.2	-	34	-	-
218.2	-	31	-	-
219.2	-	36	-	-
225.1	-	37	-	-
225.2	-	33	-	-
225.4	-	38	-	-
226.2	-	40	4	1
226.4	-	51	-	-
228.3	-	32	-	-
228.4	-	31	-	-
233.4	-	46	-	-
234.1	male	50	-	-
234.2	male	-	-	-
234.3	male	34	-	-
234.4	male	-	-	-
236.1	-	31	-	-
238.1	male	-	-	-
238.2	male	-	-	-
238.3	male	-	-	-
238.4	male	-	17	11
242.2	male	-	-	-
243.1	male	-	-	-
243.2	male	-	-	-
244.3	female	-	-	-
244.4	female	-	-	-
245.2	-	36	-	-
245.3	-	57	-	-
246.1	-	-	5	2
246.2	-	-	5	2
246.3	male	-	-	-
248.1	-	-	9	2
248.2	-	35	9	2
248.3	-	46	-	-
248.4	-	41	-	-
249.1	male	-	-	-
249.2	male	-	-	-
249.3	male	-	-	-
249.4	-	42	-	-
250.1	-	41	-	-
250.2	-	35	-	-

ID	sex	days difference from case date of presentation	case duration of stay	control duration of stay
251.2	-	42	-	-
251.4	-	38	-	-
253.1	male	-	-	-
253.2	male	-	-	-
253.3	-	31	-	-
253.4	male	33	-	-
254.1	female	-	-	-
254.2	female	-	-	-
254.3	female	-	-	-
254.4	female	39	-	-
255.4	female	-	-	-
262.1	male	-	-	-
262.2	male	-	-	-
262.3	male	-	-	-
262.4	male	-	-	-
263.1	female	-	-	-
263.2	female	-	-	-
263.3	female	-	-	-
263.4	female	-	-	-
266.1	female	41	5	1
266.2	-	36	-	-
267.1	-	-	4	2
267.2	-	-	4	1
267.3	-	-	4	6
267.4	male	37	4	2
268.1	female	34	-	-
268.2	-	39	-	-
270.1	-	48	-	-
270.2	-	43	5	2
270.3	female	-	-	-
270.4	-	42	-	-
271.1	male	39	-	-
271.2	-	45	-	-
271.4	male	32	-	-

Necessary changes in matching to identify four controls for cases. The search was expanded to any sex, controls sex is listed, date of persentation maximum +- 2 months, listed as difference in days to case date of presentation, and any length of stay, listed with case and control length of stay.

eTable 3. repeat visits due to infectious diseases

measles cases (n patients = 90, n repeat visits due to infectious diseases = 212)

ICD-Code	Description	N	% of repeat visits	Comm-ulative %
J06.9	Acute upper respiratory infection, unspecified	22	10%	10%
A09.9	Other gastroenteritis and colitis, unspecified	18	8%	19%
A09.0	Infectious gastroenteritis and colitis, unspecified	16	8%	26%
B34.9	Viral infection, unspecified	13	6%	33%
J20.9	Acute bronchitis, unspecified	13	6%	39%
H66.9	Otitis media, unspecified	9	4%	43%
J03.0	Acute streptococcal tonsillitis, unspecified	9	4%	47%
J02.9	Acute pharyngitis, unspecified	6	3%	50%
J03.9	Acute tonsillitis, unspecified	6	3%	53%
J38.5	Laryngeal spasm	6	3%	56%
J40	Bronchitis, not specified as acute or chronic	5	2%	58%
A08.4	Viral intestinal infection, unspecified	4	2%	60%
B00.2	Herpesviral gingivostomatitis and pharyngotonsillitis	4	2%	62%
J00	Acute nasopharyngitis [common cold]	4	2%	64%
L03.01	Cellulitis and acute lymphangitis of finger	4	2%	66%
N48.1	Balanitis	4	2%	67%
R50.9	Fever, unspecified	4	2%	69%

infectious disease controls (n patients = 216, n repeat visits due to infectious diseases = 464)

ICD-Code	Description	N	% of repeat visits	Comm-ulative %
J06.9	Acute upper respiratory infection, unspecified	76	16%	16%
B34.9	Viral infection, unspecified	50	11%	27%
J20.9	Acute bronchitis, unspecified	40	9%	36%
A09.9	Other gastroenteritis and colitis, unspecified	36	8%	44%
H66.9	Otitis media, unspecified	23	5%	48%
J03.9	Acute tonsillitis, unspecified	23	5%	53%
J38.5	Laryngeal spasm	19	4%	58%
A09.0	Infectious gastroenteritis and colitis, unspecified	19	4%	62%
B08.4	Enteroviral vesicular stomatitis with exanthem	11	2%	64%
J02.9	Acute pharyngitis, unspecified	9	2%	66%
J03.0	Acute streptococcal tonsillitis, unspecified	6	1%	67%
H10.0	Mucopurulent conjunctivitis	6	1%	69%
J18.0	Bronchopneumonia, unspecified organism	6	1%	70%
B00.2	Herpesviral gingivostomatitis and pharyngotonsillitis	6	1%	71%
J40	Bronchitis, not specified as acute or chronic	5	1%	72%
J18.9	Pneumonia, unspecified organism	5	1%	73%
J00	Acute nasopharyngitis [common cold]	4	1%	74%

non-infectious disease controls (n patients = 168, n repeat visits due to infectious diseases = 311)

ICD-Code	Description	N	% of repeat visits	Comm-ulative %
J06.9	Acute upper respiratory infection, unspecified	51	16%	16%
B34.9	Viral infection, unspecified	30	10%	26%
A09.9	Other gastroenteritis and colitis, unspecified	26	8%	34%
H66.9	Otitis media, unspecified	15	5%	39%
J02.9	Acute pharyngitis, unspecified	12	4%	43%
J38.5	Laryngeal spasm	12	4%	46%
J00	Acute nasopharyngitis [common cold]	12	4%	50%
J03.9	Acute tonsillitis, unspecified	10	3%	54%
J20.9	Acute bronchitis, unspecified	9	3%	56%
J03.0	Acute streptococcal tonsillitis, unspecified	8	3%	59%
A09.0	Infectious gastroenteritis and colitis, unspecified	6	2%	61%
B08.4	Enteroviral vesicular stomatitis with exanthem	6	2%	63%
H10.9	Unspecified conjunctivitis	6	2%	65%
H65.0	Acute serous otitis media	6	2%	67%
J40	Bronchitis, not specified as acute or chronic	5	2%	68%
H66.0	Acute suppurative otitis media	5	2%	70%
N39.0	Urinary tract infection, site not specified	5	2%	71%

measles cases (n patients = 90, n repeat visits due to infectious diseases = 212)

ICD-Code	Description	N	% of repeat visits	Comm-ulative %
J18.0	Bronchopneumonia, unspecified organism	3	1%	71%
A08.0	Rotaviral enteritis	2	1%	72%
B08.4	Enteroviral vesicular stomatitis with exanthem	2	1%	73%
B99	Other and unspecified infectious disease	2	1%	74%
H10.9	Unspecified conjunctivitis	2	1%	75%
H65.0	Acute serous otitis media	2	1%	75%
J04.0	Acute laryngitis	2	1%	76%
J44.89	Chronic obstructive pulmonary disease, unspecified	2	1%	77%
K12.1	Other forms of stomatitis	2	1%	78%
K35.32	Acute appendicitis with abscess	2	1%	79%
L03.11	Cellulitis of lower limb	2	1%	80%
L05.0	Pilonidal cyst with abscess	2	1%	81%
L22	Diaper dermatitis	2	1%	82%
A08.1	Acute gastroenteropathy due to Norwalk agent	1	0%	83%
A08.3	Other viral enteritis	1	0%	83%
A60.0	Herpesviral infection of genital organs and urogenital system	1	0%	83%

infectious disease controls (n patients = 216, n repeat visits due to infectious diseases = 464)

ICD-Code	Description	N	% of repeat visits	Comm-ulative %
N39.0	Urinary tract infection, site not specified	4	1%	75%
J06.8	Acute upper respiratory infections of multiple and unspecifies sites	4	1%	76%
B09	Unspecified viral infection characterized by skin and mucous membrane lesions	4	1%	77%
J03.8	Acute tonsillitis due to other specified organisms	4	1%	78%
L03.01	Cellulitis and acute lymphangitis of finger	4	1%	78%
B99	Other and unspecified infectious disease	4	1%	79%
H10.9	Unspecified conjunctivitis	3	1%	80%
H65.0	Acute serous otitis media	3	1%	81%
K12.1	Other forms of stomatitis	3	1%	81%
N48.1	Balanitis	3	1%	82%
J44.89	Chronic obstructive pulmonary disease, unspecified	3	1%	83%
A38	Scarlet fever	3	1%	83%
R50.9	Fever, unspecified	3	1%	84%
H65.1	Other acute nonsuppurative otitis media	3	1%	84%
J20.5	Acute bronchitis due to respiratory syncytial virus	3	1%	85%
J20	Acute bronchitis	3	1%	86%

non-infectious disease controls (n patients = 168, n repeat visits due to infectious diseases = 311)

ICD-Code	Description	N	% of repeat visits	Comm-ulative %
J06.8	Acute upper respiratory infections of multiple and unspecifies sites	5	2%	73%
B09	Unspecified viral infection characterized by skin and mucous membrane lesions	4	1%	74%
H10.3	Unspecified acute conjunctivitis	4	1%	75%
K12.1	Other forms of stomatitis	3	1%	76%
H10.0	Mucopurulent conjunctivitis	3	1%	77%
N48.1	Balanitis	2	1%	78%
J18.0	Bronchopneumonia, unspecified organism	2	1%	79%
J44.89	Chronic obstructive pulmonary disease, unspecified	2	1%	79%
B08.5	Enteroviral vesicular pharyngitis	2	1%	80%
B80	Enterobiasis	2	1%	81%
J02.8	Acute pharyngitis due to other specified organisms	2	1%	81%
J03.8	Acute tonsillitis due to other specified organisms	2	1%	82%
J18.9	Pneumonia, unspecified organism	2	1%	82%
A38	Scarlet fever	2	1%	83%
H65.9	Unspecified nonsuppurative otitis media	2	1%	84%
J06.0	Acute laryngopharyngitis	2	1%	84%

measles cases (n patients = 90, n repeat visits due to infectious diseases = 212)

ICD-Code	Description	N	% of repeat visits	Comm-ulative %
A88.8	Other specified viral infections of central nervous system	1	0%	84%
B02.2	Other postherpetic nervous system involvement	1	0%	84%
B08.2	Exanthema subitum [sixth disease], unspecified	1	0%	85%
B08.5	Enteroviral vesicular pharyngitis	1	0%	85%
B09	Unspecified viral infection characterized by skin and mucous membrane lesions	1	0%	86%
B34.0	Adenovirus infection, unspecified	1	0%	86%
B37	Candidal stomatitis	1	0%	87%
B37.2	Candidiasis of skin and nail	1	0%	87%
B80	Enterobiasis	1	0%	88%
B86	Scabies	1	0%	88%
H00.0	Hordeolum	1	0%	89%
H10.0	Mucopurulent conjunctivitis	1	0%	89%
H10.8	Other conjunctivitis	1	0%	90%
H60.1	Cellulitis of external ear	1	0%	90%
H60.9	Unspecified otitis externa	1	0%	91%
H66.0	Acute suppurative otitis media	1	0%	91%
H67.1	Otitis media in diseases classified elsewhere	1	0%	92%

infectious disease controls (n patients = 216, n repeat visits due to infectious diseases = 464)

ICD-Code	Description	N	% of repeat visits	Comm-ulative %
B08.5	Enteroviral vesicular pharyngitis	2	0%	86%
J02.8	Acute pharyngitis due to other specified organisms	2	0%	87%
L08.9	Local infection of the skin and subcutaneous tissue, unspecified	2	0%	87%
H10.2	other acute conjungtivitis	2	0%	88%
J02.0	Streptococcal pharyngitis	2	0%	88%
J12.1	Respiratory syncytial virus pneumonia	2	0%	88%
L04.0	Acute lymphadenitis of face, head and neck	2	0%	89%
A08.0	Rotaviral enteritis	2	0%	89%
B86	Scabies	2	0%	90%
J11.1	Influenza due to unidentified influenza virus with other respiratory manifestations	2	0%	90%
K12.0	Recurrent oral aphthae	2	0%	91%
B08.3	Erythema infectiosum [fifth disease]	2	0%	91%
J04.2	Acute laryngotracheitis	2	0%	91%
J22	Unspecified acute lower respiratory infection	2	0%	92%
K05.0	Acute gingivitis	2	0%	92%
K29.7	Gastritis, unspecified	2	0%	93%
A08.4	Viral intestinal infection, unspecified	1	0%	93%

non-infectious disease controls (n patients = 168, n repeat visits due to infectious diseases = 311)

ICD-Code	Description	N	% of repeat visits	Comm-ulative %
N76.0	Acute vaginitis	2	1%	85%
A08.4	Viral intestinal infection, unspecified	1	0%	85%
B00.2	Herpesviral gingivostomatitis and pharyngotonsillitis	1	0%	86%
L03.01	Cellulitis and acute lymphangitis of finger	1	0%	86%
R50.9	Fever, unspecified	1	0%	86%
B99	Other and unspecified infectious disease	1	0%	87%
L03.11	Cellulitis of lower limb	1	0%	87%
L05.0	Pilonidal cyst with abscess	1	0%	87%
B08.2	Exanthema subitum [sixth disease], unspecified	1	0%	88%
B37.2	Candidiasis of skin and nail	1	0%	88%
H00.0	Hordeolum	1	0%	88%
H10.8	Other conjunctivitis	1	0%	89%
H67.1	Otitis media in diseases classified elsewhere	1	0%	89%
J20.8	Acute bronchitis due to other specified organisms	1	0%	89%
L08.9	Local infection of the skin and subcutaneous tissue, unspecified	1	0%	89%
A08.2	Adenoviral enteritis	1	0%	90%
B00.9	Herpesviral infection, unspecified	1	0%	90%

measles cases (n patients = 90, n repeat visits due to infectious diseases = 212)

ICD-Code	Description	N	% of repeat visits	Comm-ulative %
J02.8	Acute pharyngitis due to other specified organisms	1	0%	92%
J03.8	Acute tonsillitis due to other specified organisms	1	0%	92%
J11.1	Influenza due to unidentified influenza virus with other respiratory manifestations	1	0%	93%
J13	Pneumonia due to Streptococcus pneumoniae	1	0%	93%
J18.1	Lobar pneumonia, unspecified organism	1	0%	94%
J18.9	Pneumonia, unspecified organism	1	0%	94%
J20.5	Acute bronchitis due to respiratory syncytial virus	1	0%	95%
J20.8	Acute bronchitis due to other specified organisms	1	0%	95%
J35.0	Chronic tonsillitis	1	0%	96%
J41.1	Mucopurulent chronic bronchitis	1	0%	96%
K02.9	Dental caries, unspecified	1	0%	97%
K12.0	Recurrent oral aphthae	1	0%	97%
K20	Esophagitis	1	0%	98%
L01.0	Impetigo, unspecified	1	0%	98%
L08.9	Local infection of the skin and subcutaneous tissue, unspecified	1	0%	99%
N39.0	Urinary tract infection, site not specified	1	0%	99%
N76.2	Acute vulvitis	1	0%	100%

infectious disease controls (n patients = 216, n repeat visits due to infectious diseases = 464)

ICD-Code	Description	N	% of repeat visits	Comm-ulative %
B08.2	Exanthema subitum [sixth disease], unspecified	1	0%	93%
J20.8	Acute bronchitis due to other specified organisms	1	0%	93%
A08.2	Adenoviral enteritis	1	0%	94%
J05.0	Acute obstructive laryngitis [croup]	1	0%	94%
K29.1	Other acute gastritis	1	0%	94%
K35.2	Acute appendicitis with generalized peritonitis	1	0%	94%
L02.4	Cutaneous abscess, furuncle and carbuncle of limb	1	0%	94%
L03.02	Cellulitis and acute lymphangitis of toe	1	0%	95%
N51.2	Balanitis in diseases classified elsewhere	1	0%	95%
R56.0	Febrile convulsions	1	0%	95%
J04.0	Acute laryngitis	1	0%	95%
L22	Diaper dermatitis	1	0%	95%
A46	Erysipelas	1	0%	96%
A54.9	Gonococcal infection, unspecified	1	0%	96%
B08.8	Other specified viral infections characterized by skin and mucous membrane lesions	1	0%	96%
G00.1	Pneumococcal meningitis	1	0%	96%
H05.0	Acute inflammation of orbit	1	0%	97%

non-infectious disease controls (n patients = 168, n repeat visits due to infectious diseases = 311)

ICD-Code	Description	N	% of repeat visits	Comm-ulative %
B01	Varicella	1	0%	90%
B27.0	Gammaherpesviral mononucleosis without complication	1	0%	91%
B34.1	Enterovirus infection, unspecified	1	0%	91%
B38	Coccidioidomycosis	1	0%	91%
B85.0	Pediculosis due to Pediculus humanus capitis	1	0%	92%
H10	Conjungtivitis	1	0%	92%
H10.2	other acute conjungtivitis	1	0%	92%
H65.1	Other acute nonsuppurative otitis media	1	0%	93%
J02	Acute pharyngitis	1	0%	93%
J02.0	Streptococcal pharyngitis	1	0%	93%
J05.0	Acute obstructive laryngitis [croup]	1	0%	94%
J12.1	Respiratory syncytial virus pneumonia	1	0%	94%
J15.7	Pneumonia due to Mycoplasma pneumoniae	1	0%	94%
J18.8	Other pneumonia, unspecified organism	1	0%	95%
K04.7	Periapical abscess without sinus	1	0%	95%
K29.1	Other acute gastritis	1	0%	95%
K35.2	Acute appendicitis with generalized peritonitis	1	0%	96%

measles cases (n patients = 90, n repeat visits due to infectious diseases = 212)

ICD-Code	Description	N	% of repeat visits	Comm-ulative %
N76.88	Other specified inflammation of vagina and vulva	1	0%	100%

infectious disease controls (n patients = 216, n repeat visits due to infectious diseases = 464)

ICD-Code	Description	N	% of repeat visits	Comm-ulative %
H16.0	Corneal ulcer	1	0%	97%
H65	Acute nonsuppurative otitis media	1	0%	97%
H65.4	Other chronic nonsuppurative otitis media	1	0%	97%
H70.0	Acute mastoiditis	1	0%	97%
J03	Acute tonsillitis	1	0%	98%
J15.9	Unspecified bacterial pneumonia	1	0%	98%
J21.0	Acute bronchiolitis due to respiratory syncytial virus	1	0%	98%
K35	Acute appendicitis	1	0%	98%
K35.8	Unspecified acute appendicitis	1	0%	98%
L03.10	Cellulitis of upper limb	1	0%	99%
N30.0	Acute cystitis	1	0%	99%
N30.9	Cystitis, unspecified	1	0%	99%
N45.9	Orchitis, epididymitis and epididymoorchitis without abscess	1	0%	99%
N70.0	Acute salpingitis and oophoritis	1	0%	100%
N70.9	Salpingitis and oophoritis, unspecified	1	0%	100%
O23.0	Infections of kidney in pregnancy	1	0%	100%

non-infectious disease controls (n patients = 168, n repeat visits due to infectious diseases = 311)

ICD-Code	Description	N	% of repeat visits	Comm-ulative %
K36	Other appendicitis	1	0%	96%
L02.2	Cutaneous abscess, furuncle and carbuncle of trunk	1	0%	96%
L02.4	Cutaneous abscess, furuncle and carbuncle of limb	1	0%	96%
L02.8	Cutaneous abscess, furuncle and carbuncle of other sites	1	0%	97%
L03.02	Cellulitis and acute lymphangitis of toe	1	0%	97%
L03.2	Cellulitis of face	1	0%	97%
L04.0	Acute lymphadenitis of face, head and neck	1	0%	98%
N51.2	Balanitis in diseases classified elsewhere	1	0%	98%
N61	Inflammatory disorders of breast	1	0%	98%
R50.80	Fever of unknown origin	1	0%	99%
R56.0	Febrile convulsions	1	0%	99%

ICD-Codes of repeat visits due to infectious diseases in measles cases, infectious disease controls and non-infectoous disaese controls, sorted by quantity in each group.

eFigure 1. Distribution of studied measles cases over Berlin

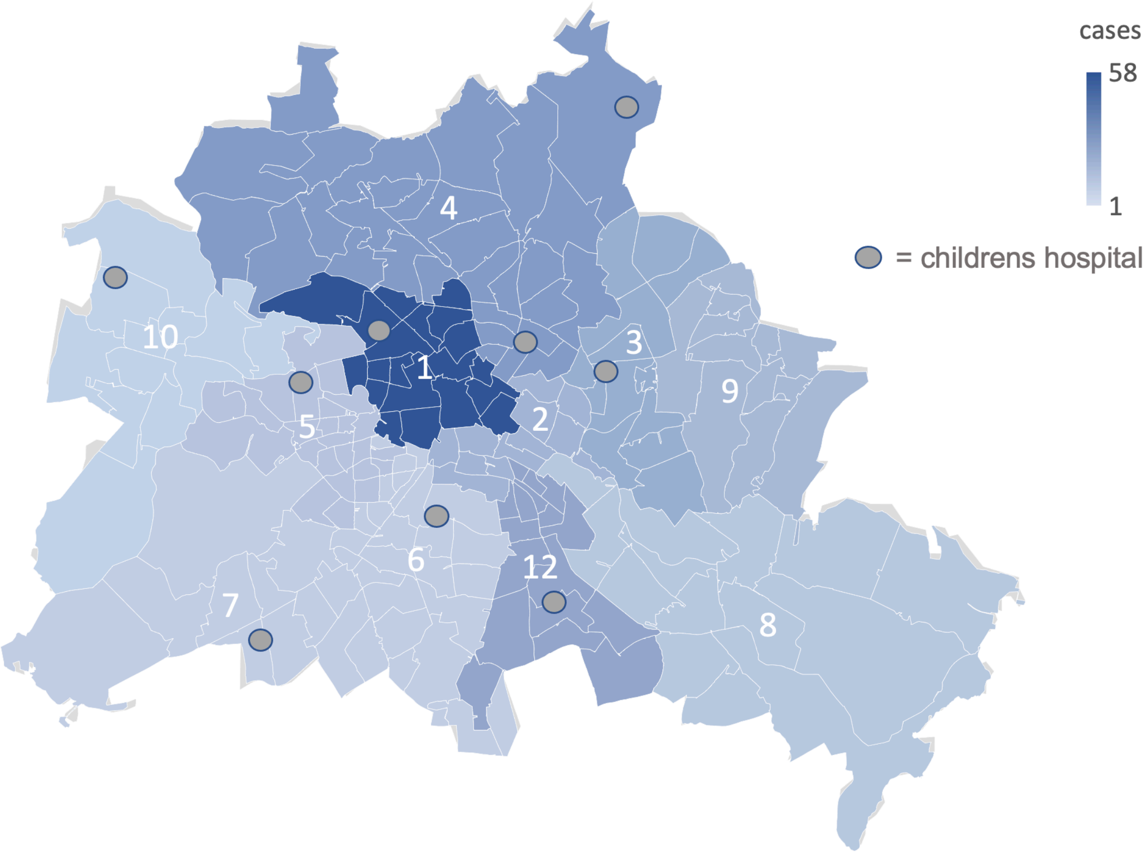

Nr. on map	district	Nr. of cases
1	Mitte	58
2	Friedrichshain-Kreuzberg	19
3	Lichtenberg	22
4	Pankow	29
5	Charlottenburg-Wilmersdorf	13
6	Tempelhof-Schöneberg	9
7	Steglitz-Zehlendorf	9
8	Treptow-Köpenick	12
9	Marzahn-Hellersdorf	17
10	Spandau	8
11	Reinickendorf	28
12	Neukölln	25

eFigure 2. Empirical cumulative distribution of age

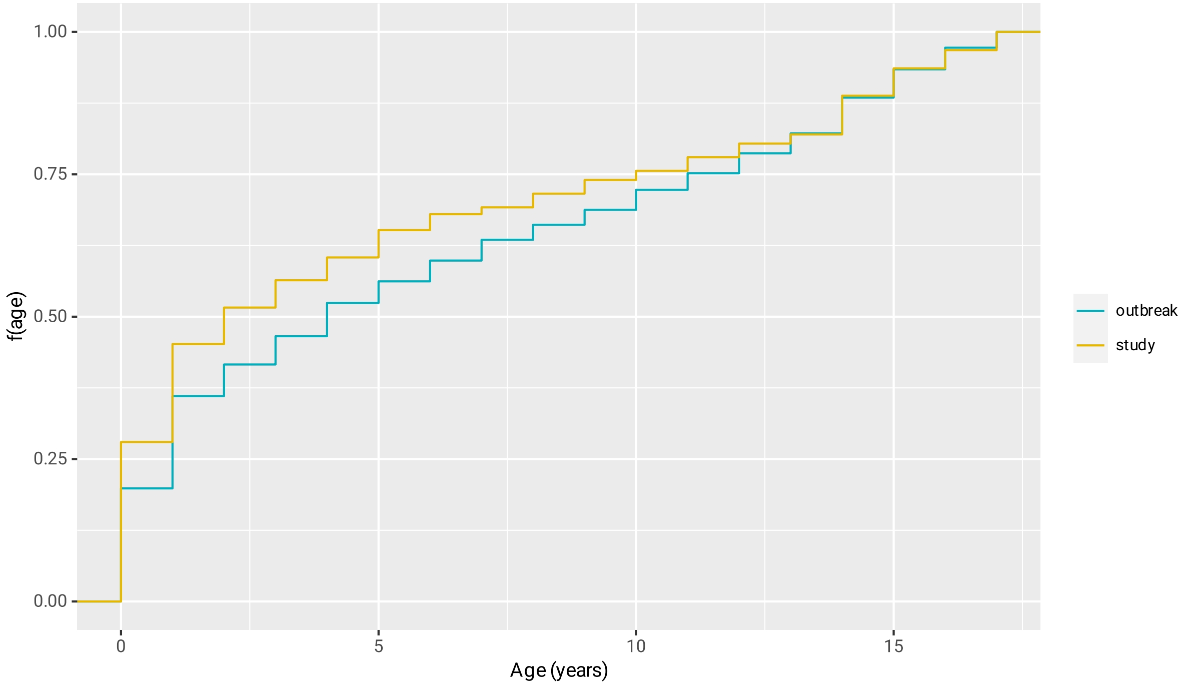

eFigure 3. Registered inhabitants per district

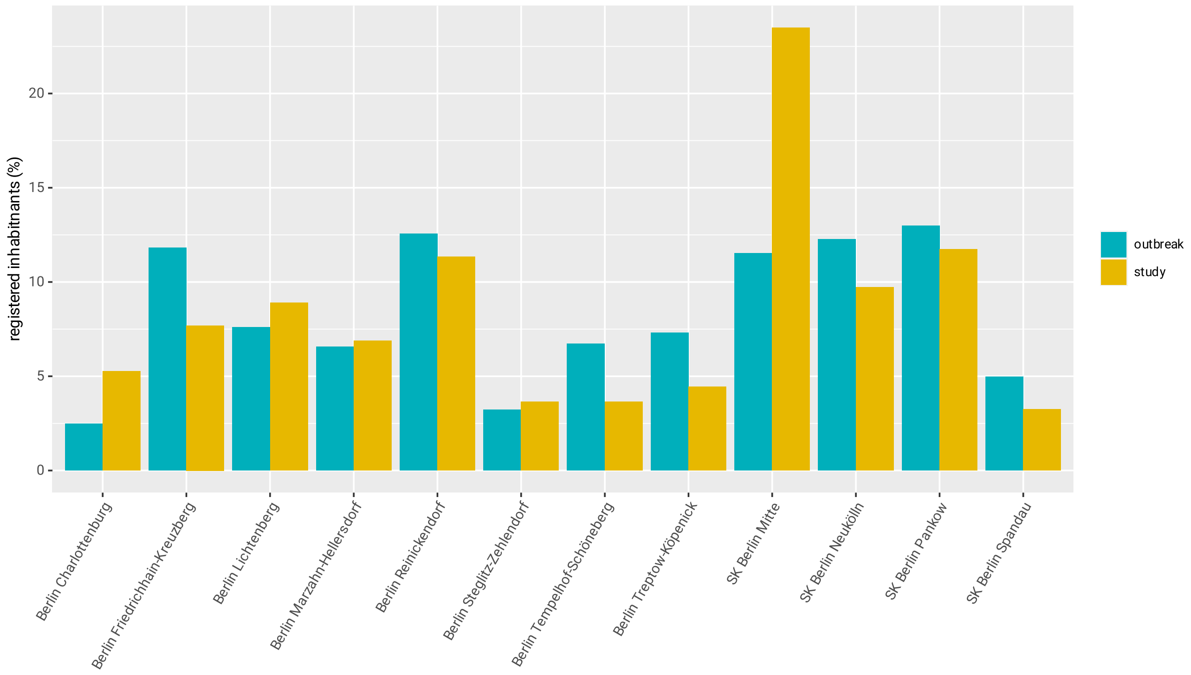

eFigure 4. Number of infectious repeated visits per patient in percent per group

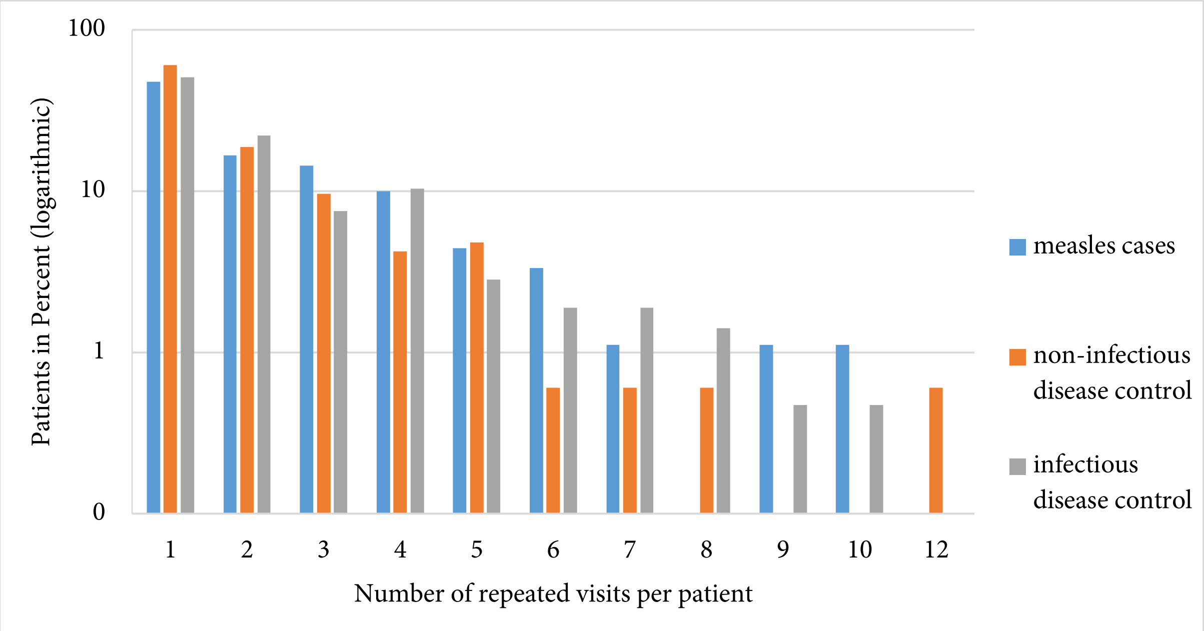

measles cases = blue, non-infectious control = orange, infectious control = gray
